# Integrated Cobaloxime and Mesoporous Silica-Supported Ruthenium/Diamine Co-Catalysis for One-Pot Hydration/Reduction Enantioselective Sequential Reaction of Alkynes

**DOI:** 10.3389/fchem.2021.732542

**Published:** 2021-09-22

**Authors:** Zeyang Liu, Yongjie Wang, Kaihong Liu, Shanshan Wang, Haocheng Liao, Yuanli Zhu, Baoming Hou, Chunxia Tan, Guohua Liu

**Affiliations:** Key Laboratory of Resource Chemistry of Ministry of Education, Shanghai Key Laboratory of Rare Earth Functional Materials, Shanghai Normal University, Shanghai, China

**Keywords:** asymmetric catalysis, heterogeneous catalysts, hydration, transfer hydrogenation, silica

## Abstract

This study developed a cost-efficient hydration/asymmetric transfer hydrogenation (ATH) process for the one-pot synthesis of valuable chiral alcohols from alkynes. During this process, the initial homogeneous cobaloxime-catalyzed hydration of alkynes was followed by heterogeneous Ru/diamine-catalyzed ATH transformation of the *in-situ* generated ketones, which provided varieties of chiral alcohols in good yields with up to 99% *ee* values. The immobilized Ru/diamine catalyst could be recycled at least three times before its deactivation in the sequential reaction system. This work shows a general method for developing one-pot asymmetric sequential catalysis towards sustainable organic synthesis.

## Introduction

Exploiting economic and environmentally friendly methodologies for multi-step sequential asymmetric organic transformation is of considerable importance in modern synthetic chemistry but presents great challenges (Climent et al., 2011; Climent et al., 2014; Van Oers et al., 2014; Parlett et al., 2016; Szőllősi, 2018; Usui et al., 2019; Cheng et al., 2020). Enantiopure alcohols are essential building blocks for a wide range of pharmaceuticals and agrochemicals (Alonso et al., 2004; Beller et al., 2004). Due to the large number of commercially available alkyne substrates (Chinchilla, and Nájera, 2014; Thomas et al., 2014; Habrant et al., 2010; Ramesh et al., 2021), the direct conversion of these alkynes into valuable optically pure alcohols is highly desirable. A cascade hydration/reduction of alkynes is a common strategy used in this atomic economic synthetic route. Recently, several typical examples of the one-pot synthesis of chiral alcohols from alkynes (Supplementary Table S3) has been reported, mainly focusing on the combination of different types of hydrations and enantioselective reductions. These include the use of excess formic acid as a solvent-mediated hydration coupled with Rh-catalyzed asymmetric transfer hydrogenation (ATH), (Li et al., 2013), noble metallic/ligand catalysts such as [(iPr)AuX] (X = NTf_2_ or BF_4_), (Ye et al., 2015; Xia et al., 2017), [(iPr)AuCl], (Li et al., 2015), Co-Salen (Wang et al., 2014), and Co-Porphyrin (Lu et al., 2015) mediated hydration coupled with Rh-catalyzed ATH, and TfOH-catalyzed hydration coupled with Rh-mediated asymmetric hydrogenation (AH) (Liu et al., 2018a) or ATH (Liu et al., 2018b). Despite the great developments that have been made in the one-pot synthesis of enantiopure alcohols from alkynes, most of the reactions should be conducted under high temperatures (Li et al., 2013), high pressure, (Liu et al., 2018a), and/or in acidic media which require a large amount of NaOH for pH adjustment. Furthermore, the expensive cost of metals and ligands is still a barrier to the preparation of corresponding catalysts in gram-scale. Therefore, exploiting a more efficient and economical catalytic system for the one-pot synthesis of chiral alcohol from the commercially available alkynes under mild reaction conditions has great significance for practical applications.

**GRAPHIC ABSTRACT:**
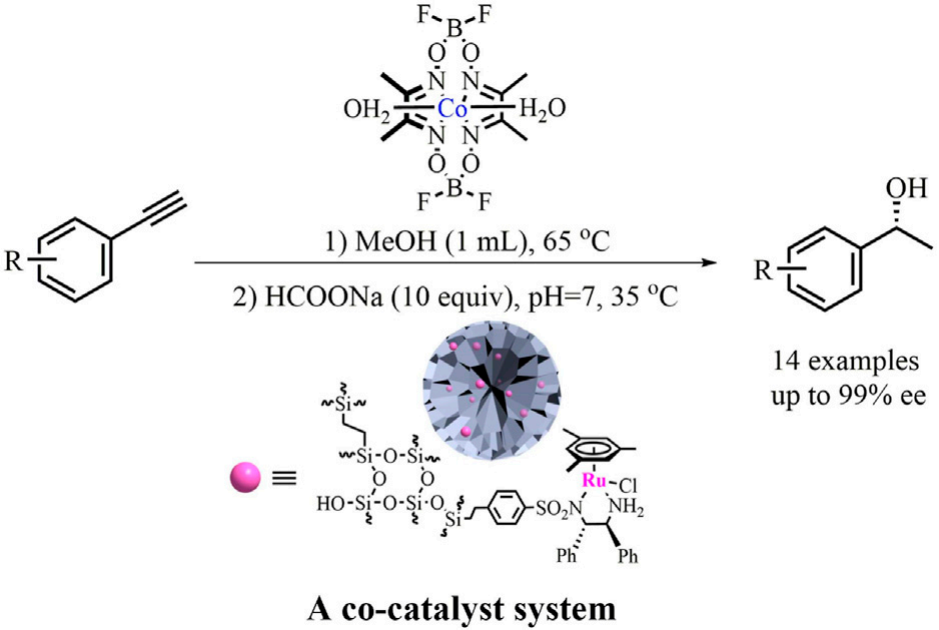
One‐Pot Hydration/Reduction Enantioselective Sequential Reaction of Alkynes

Immobilization of chiral organometallic complexes onto the specific skeleton mesoporous silica materials [such as FDU-12 (Gao, et al., 2013), SBA-15 (Long, et al., 2013), and KCC-1 (Polshettiwar, et al., 2010; Fihri, et al., 2012), etc] has been extensively used in the construction of heterogeneous chiral catalysts, wherein several well-established strategies have been applied to the recyclable synthesis of various optically active compounds (De Vos et al., 2002; Yang et al., 2007; Ding, and Uozumi, 2008; Minakata, and Komatsu, 2009; Bartók, 2010; Mehdi et al., 2011). In particular, some multifunction materials have shown superiority in the construction of heterobifunctional catalysts, which enable a highly efficient cascade process including dynamic kinetic resolution/ATH, allylic alkylation/Pauson–Khand annulation, Knoevenagel condensation/hydrogenation reactions, Suzuki/Heck reactions, and Sonogashira-Henry reactions (Climent et al., 2014) As a kind of unique silica support, the dendritic mesoporous organosilica nanoparticles (DMONs) have a central-radical pore structure (Melde, et al., 1999; Linares, et al., 2012). This feature means they possess a large pore size and highly accessible surface areas. This advantage not only acts as a storage reservoir for catalysts and guest molecules but also enables an efficient mass transfer in the hollow, thereby providing a promising platform for catalysis application. Therefore, it is reasonable to expect that the combination of an inexpensive organometallic complex and a chiral Ru/diamine enables an efficient and recyclable hydration/ATH cascade process that has still not been explored.

Compared to the Au/carbine-complexes used in the hydration of alkynes, cobaloximes [Co(dmgBF_2_)_2_·2H_2_O, dmg = dimethylglyoximate] are cost-efficient since this strong Lewis acid catalyst with bench stability could be easily prepared on a large scale from cheap raw materials. This is especially true as it is also an efficient hydration catalyst for a wide range of terminal alkynes under neutral conditions (MeOH, 65°C, air) (Bartók, 2010). This superiority offers a practical opportunity for the one-pot hydration/reduction of alkynes to overcome the limitations of environmental issues, high cost, and/or the harsh conditions originating from noble metal/ligand catalysts. Due to the benefits of the compatibility of the mild catalysis condition of cobaloximes with the Rh/diamine catalyst, and taking into account our recent progress in silica-based chiral recyclable heterogeneous catalyst through a covalent-bonding method (Chang et al., 2020; Li et al., 2021; Zhao et al., 2021), we envision that *via* a one-pot hydration/ATH catalyzed with a combination of inexpensive cobaloximes (Schrauzer, and Windgassen, 1967; Bakac et al., 1986; Geno, and Halpern, 1987; Hou et al., 2017) and DMONs-based Rh/diamine as co-catalysts (Hashiguchi et al., 1995; Hannedouche et al., 2004; Matharu et al., 2005; Hayes et al., 2005; Ohkuma et al., 2006; Cheung et al., 2007; Touge et al., 2011; Touge et al., 2016), the alkynes could be converted into chiral alcohols. As presented in this study, this sequential enantioselective organic transformation, an initial homogeneous cobaloxime-catalyzed hydration of alkynes followed by a subsequent heterogeneous Ru/diamine-catalyzed ATH transformation of *in-situ* generated ketones, provided various chiral alcohols in good yields and up to 99% *ee*.

## Experiment

### Preparation of the Catalysts

Co(dmgBF_2_)_2_·2H_2_O (catalyst 1) was synthesized according to the method outlined in the literature method (Hou et al., 2017). First, 150 ml degassed diethyl ether was added into a 500 ml round-bottomed flask containing 2.0 g [Co(OAc)_2_·4H_2_O] (8 mmol, 1 eq.) and 1.0 g dmgH_2_ (1.9 g, 16 mmol, 2 eq.) under argon atmosphere, then 10 ml freshly distilled BF_3_·Et_2_O was added. The mixture was stirred for 6 h at room temperature. The brownish-red solid was then filtered under argon and washed with degassed ice-cold water (3 × 10 ml), then dried at 60 °C under vacuum overnight, and the target solid product was obtained in a 60% yield (2.0 g, 4.8 mmol). IR (KBr, cm^−1^) (Supplementary Figure S1): 3,601, 3,530, 3,023, 2,964, 2,927, 1,622, 1,572, 1,438, 1,385, 1,307, 1,287, 1,249, 1,164, 1,147, 1,098, 1,084, 1,011, 962, 831, 630, and 608. LC-MS (Supplementary Figure S2): m/z 422.0597 {Calcd m/z 422.0602 for [Co.(dmgBF_2_)_2_·2H_2_O+ H]^+^}. UV-Vis (Supplementary Figure S3, DMSO, 6 × 10^−5^ M, 2.5 cm quartz cell): 446, 335 nm^2^. ^1^H NMR (400 MHz, DMSO-*d*_6,_ Supplementary Figure S4) *δ* 3.17 (s, 1H). ^13^C NMR (101 MHz) *δ* 186.75, 18.35.

**SCHEME 1:**
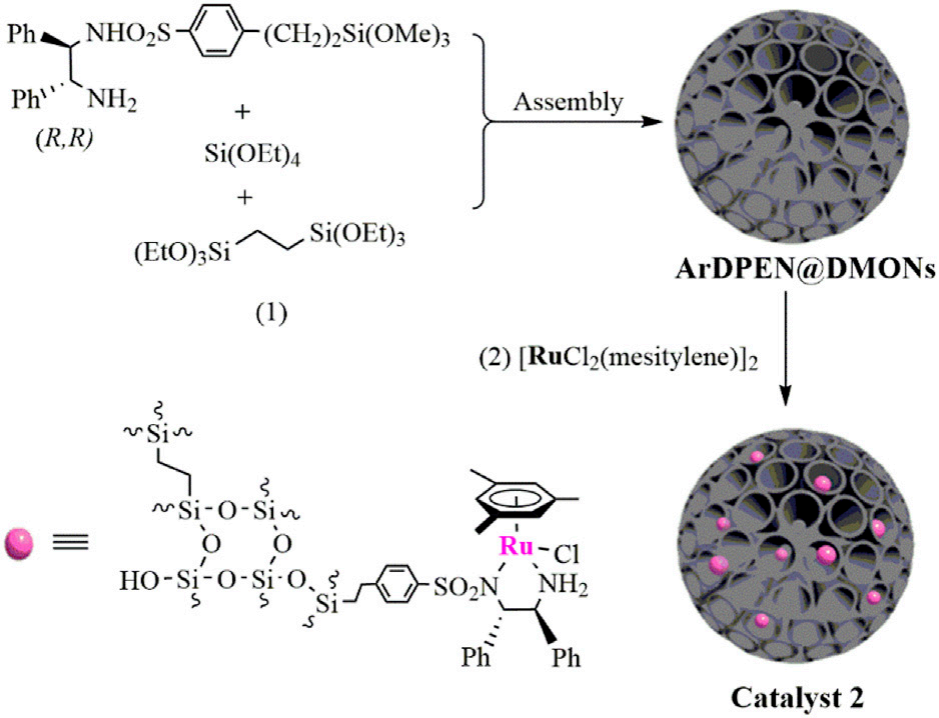
Synthesis of the catalysts 2.

Immobilization chiral catalytically active centers into the dendritic mesoporous organosilica nanoparticles to prepare a Ru/diamine–functionalized heterogeneous catalyst, abbreviated as MesityleneRuArDPEN@DMONs (catalyst 2) (ArDPEN = (*R, R*)-4-((trimethoxysilyl)ethyl)phenylsulfonyl-1,2-diphenylethylene-diamine), were synthesized in a typical two-step procedure (Scheme 1) (Hashiguchi et al., 1995; Fujii et al., 1996; Liu et al., 2003; Wu et al., 2004; Fan et al., 2005; Hayes et al., 2005; Ikariya, and Blacker, 2007; Ohkuma et al., 2007; Ma et al., 2010; Yang et al., 2016). The first step is the synthesis of the dendritic mesoporous organosilica nanoparticles. This involved dissolving 0.068 g triethanolamine in 25 ml H_2_O and stirred at 1,000 rpm under 80°C. Then the structure-directing reagents were added to cetyltrimethylammonium bromide (CTAB) and sodium salicylate (NaSal) after 30 min, and then the mixture was stirred for 1 h. After reducing the stirring speed to 300 rpm, the mixture of TEOS (tetraethyl orthosilicate, 2.0 ml) and BTEE (1,2-bis(triethoxysilyl)ethane, 1.6 ml) was injected into the above solution and stirring was continued for 9 h. Afterward, 2 ml ethanol solvent containing 1,2-bis(triethoxysilyl)ethane (0.89 g, 2.5 mmol) and (*R, R*)-ArDPEN-siloxane (0.15 g, 0.3 mmol) was added, and the mixture was stirred for another 3 h. Then the dendritic mesoporous organosilica nanoparticles ((*R, R*)-ArDPEN@DMONs) were collected after centrifugation and washed with ethanol (30 ml × 3). To remove the template, the obtained (*R, R*)-ArDPEN@DMONs were immersed in the HCl/methanol mixture solution (30 ml, v/v = 1/3) and stirred at 60.0°C for 6 h three times. The obtained solid was dried in a vacuum at 60°C overnight. For the second step, Ru/diamine–functionalized heterogeneous catalyst (catalyst 2) was synthesized by adding (MesityleneRuCl_2_)_2_ (50.0 mg, 0.097 mmol) into a suspension of (*R, R*)-ArDPEN@DMONs (0.50 g) in dry CH_2_Cl_2_ (20.0 ml) at room temperature. The mixture was stirred at 25°C for 12 h. The mixture was then filtered and washed by dry CH_2_Cl_2_ several times. The target catalyst 2 was collected as a light-brown powder after being dried at 60°C under a vacuum overnight. The ICP analysis indicated that the content of Ru was 5.255 mg (0.052 mmol) per gram of heterogeneous catalyst. ^13^C CP/MAS NMR (161.9 MHz): 137.3–116.7 (*C* of Ar and Ph groups), 106.3 (*C* of Arene group), 72.4–68.4 (*C* of −N*C*HPh), 36.8–27.4 (*C* of–*C*H_2_Ar), 20.8–12.7 (*C* of–Si*C*H_2_ *C*H_2_Si, and *C* of −*C*H_2_Si), 21.4–28.8 (*C* of Arene*C*H_3_, 5.1) ppm. ^29^Si MAS NMR (79.4 MHz): T^2^ (*δ* = −58.5 ppm), T^3^ (*δ* = −68.4 ppm), Q^3^ (*δ* = −102.7 ppm), Q^4^ (*δ* = −111.9 ppm). Elemental analysis: *C 11.30, H 2.56, N 0.32, S 0.37.* IR (KBr, cm^−1^): 3,423, 3,058, 3,013, 2,927, 1966, 1868, 1,622, 1,521, 1,497, 1,455, 1,409, 1,378, 1,327, 1,150, 1,092, 925, 797, 700, 634, 522, and 464.

### General Procedure for the Hydration-ATH One-Pot Enantioselective Sequential Reaction

In a 10.0 ml round-bottom flask, the catalyst 1 (2.0 mol%), alkyne (0.25 mmol), and methanol (1.0 ml) were added sequentially. The mixture was stirred at 65°C under aerobic conditions for 2.5–5 h. After the hydrolysis was completely determined by TLC, it was quenched with 1.0 ml H_2_O and the pH was adjusted to 7 with 3 drops of 0.4 mol/L NaOH. Consequently, the heterogeneous catalyst 2 (20 mg, 1.04 µmol of Ru (0.4 mol%), based on ICP analysis) and HCOONa (170 mg, 10 equiv) were added under stirring, then the ATH reaction was maintained at 35°C for 12 h until acetophenone was transformed to phenylethanol, completely determined by TLC. The mixture was separated by centrifugation (10,000 rpm), and the aqueous solution was extracted with ethyl acetate (3 × 3.0 ml). The combined organic layer was dried over MgSO_4_ and evaporated in vacuo. The product was further purified by a flash silica gel column to afford the desired product (EA/PE = 1/15). The ee values were determined by HPLC analysis with a UV-Vis detector and a Daicel chiralcel column (*Φ* 0.46 × 25 cm).

### Procedure for the Recycle of Catalyst 2

Catalyst 1 (2.0 mol%), alkyne (0.25 mmol), and methanol (1.0 ml) was added sequentially to a 10.0 ml round-bottom flask. The mixture was then stirred at 65°C under aerobic conditions for 5 h. The reaction was monitored by TLC to confirm the completion of the hydration, then quenched with 1.0 ml H_2_O and the pH adjusted to 7 with 3 drops of 0.4 mol/L NaOH. Consequently, the heterogeneous catalyst 2 [20 mg, 1.04 µmol of Ru (0.4 mol%), based on ICP analysis], and HCOONa (170 mg, 10 equiv) were added, and the mixture stirred at 35°C for 12 h. After completion of the reaction determined by TLC, the mixture was centrifuged at 10,000 rpm for 5 min, and the precipitate was Soxhlet extracted with methanol and DCM until no catalyst 1 and product was detected in the eluent. The recovered solid was reactivated at 60°C under vacuum overnight and then reused for the next runs directly.

## Results and Discussion

### Synthesis and Structural Characterization

**FIGURE 1:**
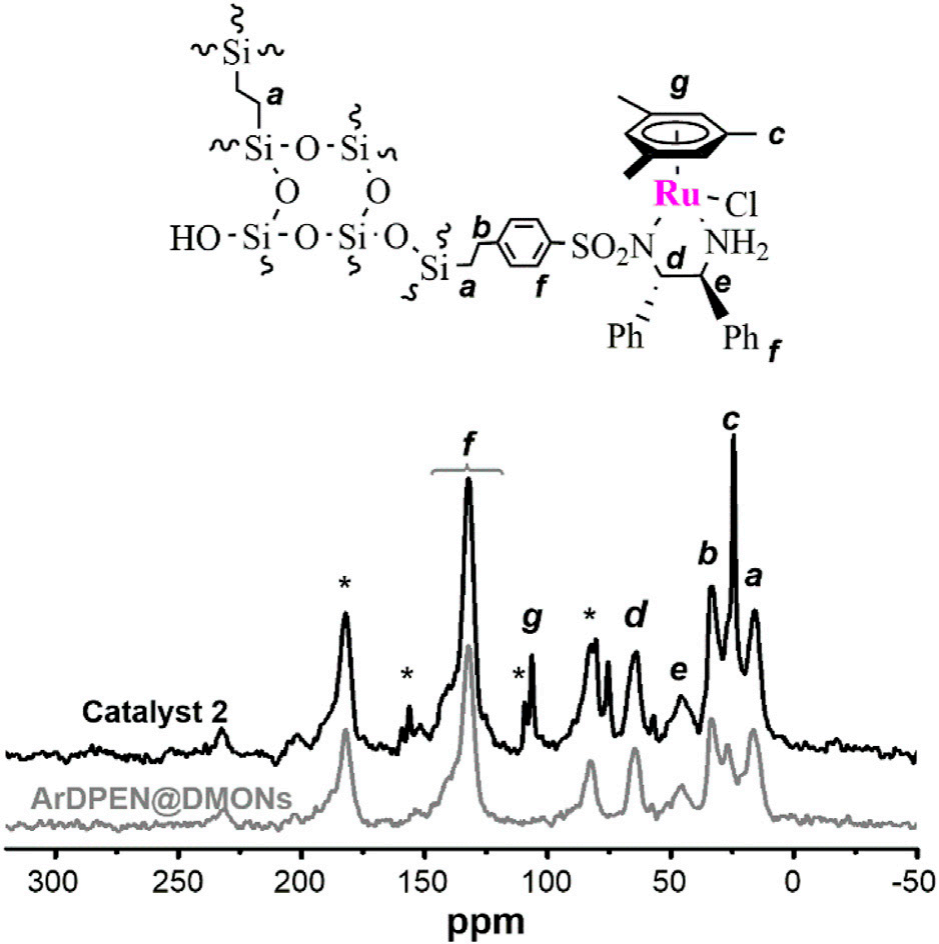
Solid-state ^13^C CP/MAS NMR spectra of ArDPEN@DMONs and catalyst 2.

**FIGURE 2:**
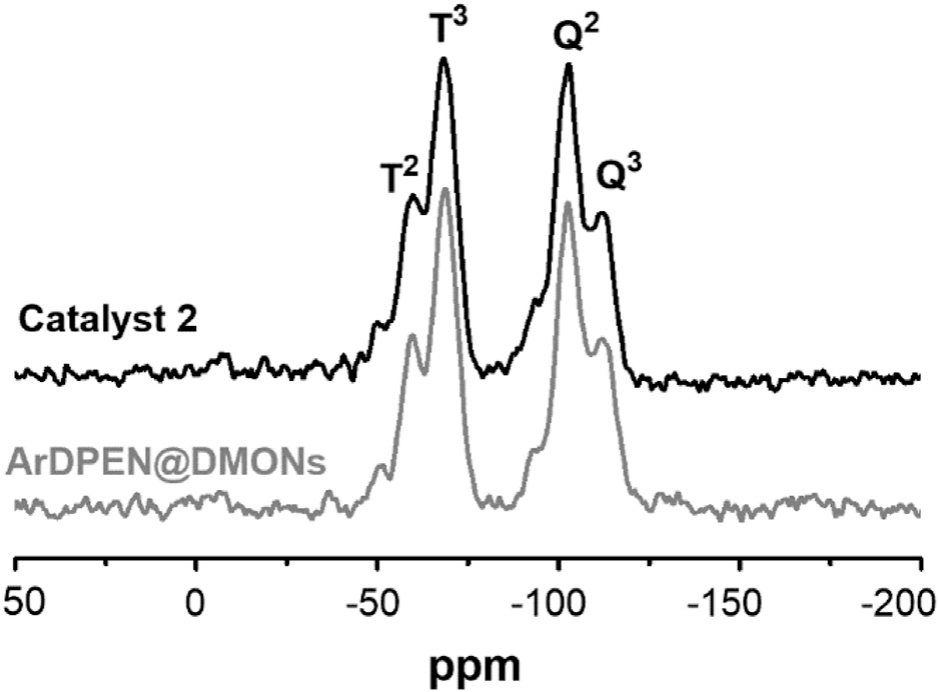
Solid-state ^29^Si CP/MAS NMR spectra of ArDPEN@DMONs and catalyst 2, *The rotation band.

The solid-state ^13^C cross-polarization (CP)/magic angle spinning (MAS) NMR spectroscopy was detected to confirm the chiral ruthenium/diamine species had been incorporated within the dendritic mesoporous organosilica nanoparticles of (*R, R*)-ArDPEN@DMONs. As shown in Figure 1, the strong carbon signals of–Si*C*H_2_ *C*H_2_Si–moiety around 15 ppm was produced, which corresponded to the ethyl–bridged organosilica in catalyst 2. The characteristic peak ascribed the carbon atoms of the aromatic ring in the mesitylene group around 106 ppm, which has been shown, and the peaks originated from the carbon atoms of the–*C*H_3_ groups attached to the mesitylene group are around 21 ppm. Further, carbon atoms of–N*C*H groups connected to phenyl groups in ArDPEN moiety correspond to the peaks between 67 and 73 ppm. All these observed carbon signals were similar to those of its homogeneous MesityleneRuArDPEN, revealing that catalyst 2 had the same well–defined single-site active species as the MesityleneRuTsDPEN. Hashiguchi et al. (1995) For the Solid-state ^29^Si MAS NMR spectrum of catalyst 2 (Figure 2), two strong T signals around −58 and −68 ppm correspond to T^2^ {[R–Si(OSi)_2_(OH)]} and T^3^ [R–Si(OSi)_3_] (R = alkyl–species originated from linked MesityleneRuArDPEN groups or ethyl–bridged groups), demonstrating that the incorporated precursors were covalently converted within its organosilica network. The other two Q signals at -102 and -111 ppm are attributed to Q^3^ (Si(OSi)_3_(OH)) and Q^4^ (Si(OSi)_4_ species coming from TEOS precursor (Kröcher et al., 1998).

**FIGURE 3:**
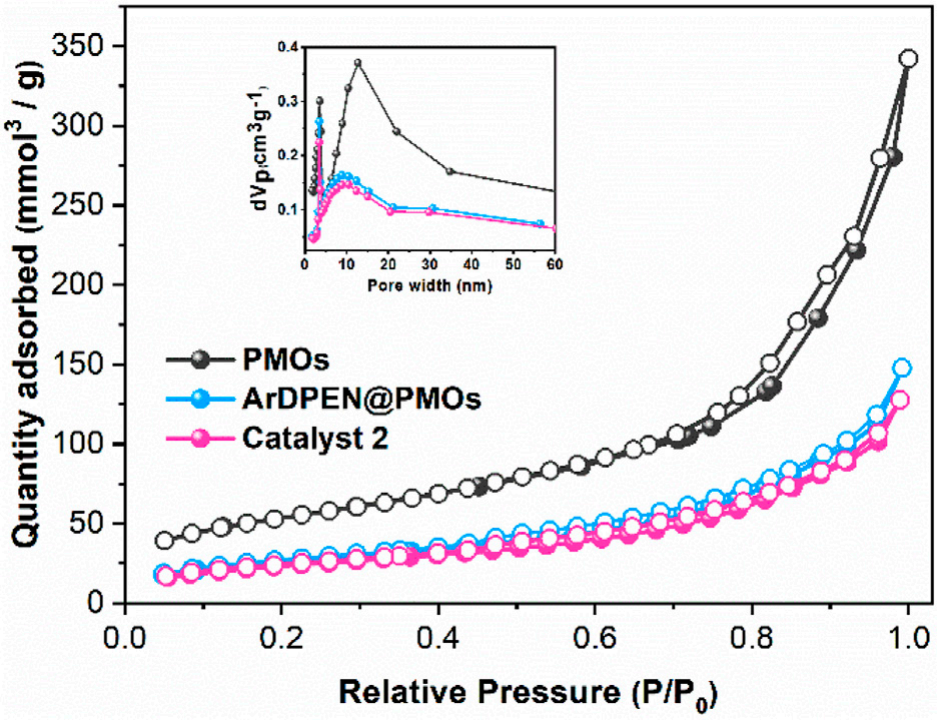
Nitrogen adsorption-desorption isotherms of PMOs, ArDPEN@DMONs, and catalyst 2.

**FIGURE 4:**
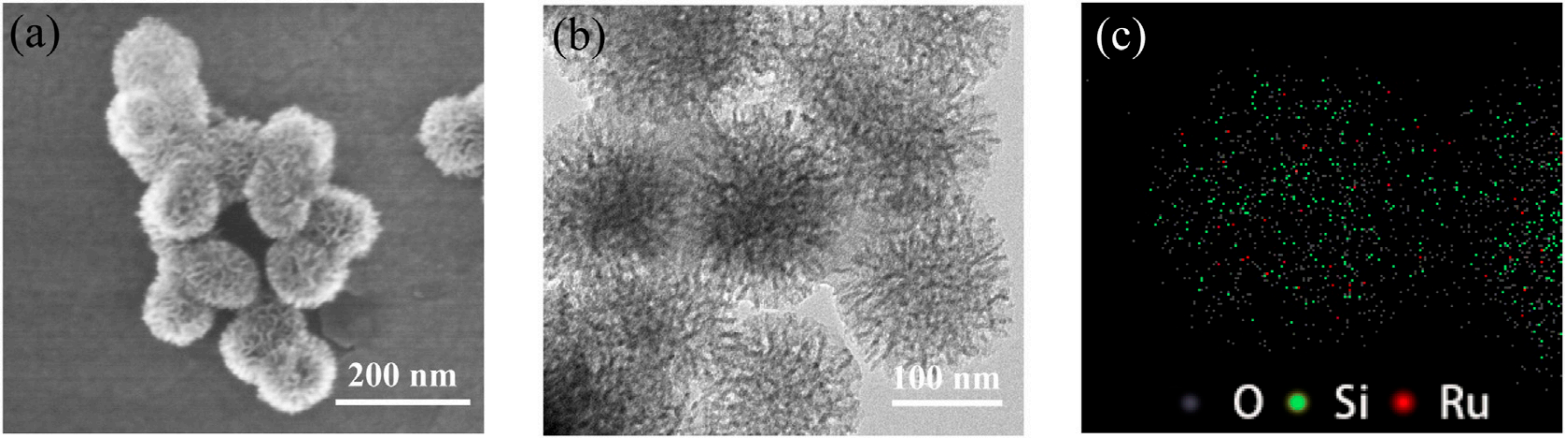
SEM image **(A)**, TEM image **(B)**, and EDS mapping **(C)** of catalyst 2.

To illustrate the pore structure, morphology, and distribution of ruthenium in catalyst 2, the nitrogen (N_2_) adsorption-desorption isotherms, scanning electron microscopy (SEM) images, and transmission electron microscopy (TEM) images were recorded. As shown in Figure 3, the nitrogen adsorption-desorption isotherms shown that ArDPEN@DMONs and catalyst 2 have the same typical type IV isotherms with an H_1_ hysteresis loop, which were similar to that of the corresponding pure DMONs materials, except for the reduced mesopore size (9.55 nm for ArDPEN@DMONs, 9.26 nm for catalyst 2 versus 11.26 nm for DMONs), surface area (95.64 m^2^/g for ArDPEN@DMONs, 85.23 m^2^/g for catalyst 2, versus 187.87 m^2^/g for DMONs), and pore volume (0.23 cm^3^/g for ArDPEN@DMONs, 0.20 cm^3^/g for catalyst 2, versus 0.53 cm^3^/g for DMONs), suggesting that the decoration of the ArDPEN and the complexation of ArDPEN@DMONs with (MesityleneRuCl_2_)_2_ led to the nanopore narrowing in the catalyst 2. The uniformly ordered pore arrangements in catalyst 2 were revealed by the SEM and TEM images as shown in Figures 4A,B, and the average size for catalyst 2 was around 100 nm. The elemental mapping for catalyst 2 at the microstructural level by TEM with energy dispersive spectra (EDS) showed uniform distribution of Ru centers within its nanochannels (Figure 4C), which further confirmed that the chiral Ru/diamine active centers were incorporated steadily within the DMONs network. Therefore, the above structural analyses and characterization indicated that the evenly distributed catalytic active site in the stable ordered dendritic mesoporous organosilica nanoparticles would govern the efficient and recyclable catalytic performance of catalyst 2.

### Hydration-Asymmetric Transfer Hydrogenation Catalysis

Chiral N-sulfonylated diamine functionalized ruthenium complexes were classic catalytically efficient active species for ATH. Notably, the reaction conditions (1 mol% Ru, 10 equiv HCOONa or HCOOH/Et_3_N as a hydrogen source, H_2_O or alcohols as the solvent) are not only simple but also partially compatible with the hydration process catalyzed by cobaloxime. The corresponding dendritic mesoporous structure can provide a hollow void space to concentrate reactants which may enhance the reactivity and enantioselectivity (Fihri, et al., 2012) relative to the free N-sulfonylated diamine functionalized ruthenium complex. Therefore, distributing the catalytic active site in the ordered DMONs would have obvious superiority in maintaining and/or even improving the catalytic activity as well as realizing the recyclable nature of the Ruthenium/diamine catalyst. Thus, the integration cobaloxime with mesoporous silica-supported Ruthenium/diamine co-catalysis for the one-pot hydration/ATH sequential reaction of alkynes indicates a newly atomic-economic, environment-friendly, and mild conditioned process.

**FIGURE 5:**
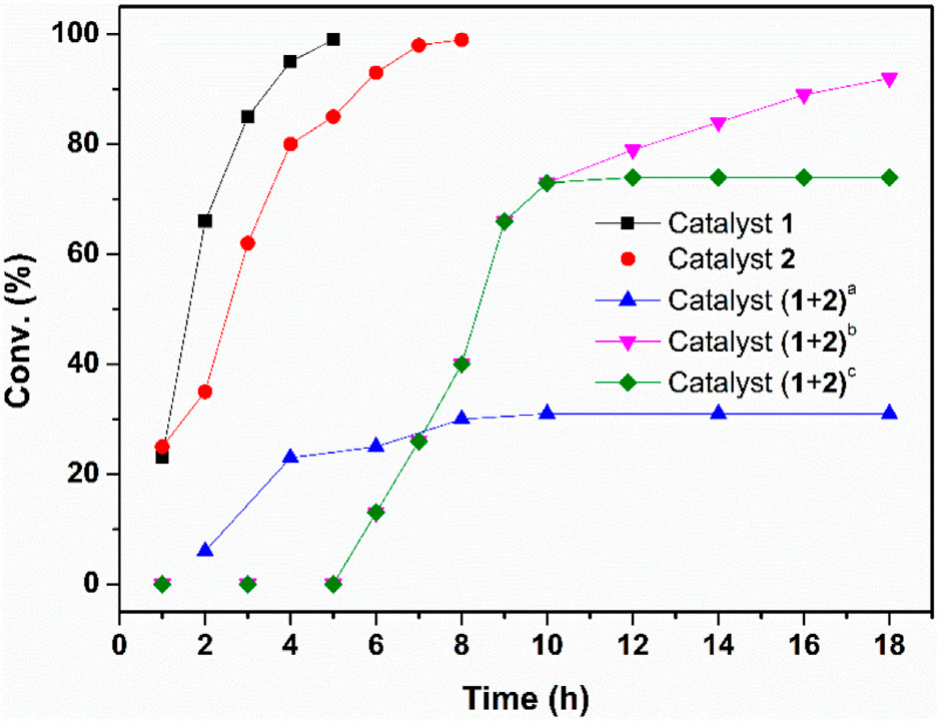
Kinetic results of the one-pot hydration-ATH sequential reaction of phenylacetylene.

Our investigation started by combining both reactions into a one-pot process. We chose enantioselective cascade hydration/ATH of phenylacetylene as a model reaction. To our delight, both catalysts 1 and 2 could transform the corresponding substrate with quantitative yield independently under aerobic conditions (entries 1-2, Supplementary Table S1). However, when the two reactions were performed under an argon atmosphere, the hydration by 1 was unresponsive but the ATH reaction performed smoothly (entries 1-5, Supplementary Table S1). This result reflects other studies in which active catalytic species Co(III) could not generate *in situ* without O_2_. (Bartók, 2010). We then explored the catalytic activity of 1 and 2 in the mixture reaction system. Results revealed that, when adding the two catalysts together under aerobic conditions, the alkyne could be transformed quantitatively but only part of acetophenone (30%) was transformed to phenylethanol (entry 6, Supplementary Table S1), whereas when the same sequential process was performed under an argon atmosphere, the hydration reaction was obstructed (entry 7, Supplementary Table S1) while the ATH reaction was processed smoothly (entry 8, Supplementary Table S1). The above results indicated that the active catalytic species Co(III) generated *in situ* is the key point to the hydration process but it deactivates the Ruthenium/diamine catalyst, leading to less efficient sequential reactions when adding the two catalysts together. Then, the reaction was attempted *via* a step-wise method, and the catalytic activities for the sequential reactions at low catalyst/substrate (C/S) ratios [the molar ratio of catalyst to alkyne] were further compared (Supplementary Table S2). It shows that when the loading was decreased from 1 to 0.5 mol% and 0.2 to 0.1 mol% for catalyst 1 and 2 respectively, the conversion decreased from 95 to 77%, indicating the catalyst content plays an important role in the highly efficient synthesis of chiral alcohols. Therefore, the general catalysis condition for this work are determined as the hydration of phenylacetylene (1a) with a 2 mol% catalyst 1 and 1.0 ml of methanol, after 1a was converted to corresponding acetophenone quantitatively at 65°C in about 2.5 h, 1.0 ml H_2_O was added to quench the hydration and 3 drops of NaOH (0.4 mol/L) were added to adjust the pH of the weak acidic mixture to neutral. Subsequently, 0.4 mol% catalyst 2, 10 equiv. of HCOONa, were added. The ATH was conducted at 35°C for 12°h, affording the desired (*R*)-1-phenylethanol (1c) up to 84% yield and 96% ee (Table 1, entry 1). The kinetic reaction profiling (Figure 5) for each step and the sequential process were compared to further clarify how the two catalysts perform during the one-pot reaction. The results revealed that adding the two catalysts together obtained the target phenylethanol at the beginning, but the activity of the catalyst 2 decreased drastically during the hydration process, and the conversion of the acetophenone stopped after hydration finished and the yield of the phenylethanol was lower (32%) compared with when the catalyst added sequentially (92%). Moreover, it showed the conversion of the acetophenone in the separated solution did not increase after the reaction mixture was filtered to remove the solid catalyst 2. These results further confirmed the deactivated activity of the Ruthenium/diamine by cobaloximes and that fewer activities of the Ru species in the solvent were leached from the catalyst 2.

**TABLE 1:** The Hydration–ATH one-pot enantioselective tandem reactions of alkynes into chiral alcohol.^a^

Entry	R	Yield (%)	ee^b^ (%)
1	H	84%	96%
2^c^	H	85%	97%
3	4-Me	85%	96%
4	4-Et	95%	99%
5	4-^t^Bu	93%	99%
6	4-MeO	84%	97%
7	3-MeO	89%	93%
8	2-MeO	89%	99%
9	4-F	93%	95%
10	4-Cl	93%	93%
11	4-Br	91%	92%
12	3-Br	93%	99%
13	4-NO_2_	74%	77%
14	Thiophene	91%	97%
15	4-alkynyl	83%	99% (de = 9:1)
16^d^	H	0	ND
17^e^	H	35%	74%
18^f^	H	41%	77%
19^g^	H	77%	87%

a Reaction conditions: 0.25 mmol of alkyne 1a, cobaloxime (2 mol%) in MeOH (1 ml), heated at 65°C under aerobic conditions, reaction time (2.5–6 h, except 60 h for 4-nitrophenylacetylene and 16 h for 2-ethynylthiophene), quenched with 1 ml of H2O; then adjust PH to 7 with 0.4 mol/L NaOH, 20 mg Catalyst 2 (20 mg, 1.04 μmol of Ru (0.4 mol%), based on ICP analysis), HCOONa (170 mg, 10 equiv), reaction temperature 35°C, reaction time 12 h.

b The ee values were determined by chiral HPLC analysis.

c Data were obtained with homogeneous counterparts as dual catalysts.

d Without NaOH solvent.

e adjust PH to 3.

f adjust PH to 12.

g adjust PH to 10.

Having established the above compatible catalytic system for the efficient hydration/ATH catalysis of alkynes into chiral alcohols, the general applicability of the one-pot enantioselective sequential catalysis system was further investigated with a series of substituted substrates. As shown in Table 1, most of the tested alkyne substrates could be smoothly transformed into the corresponding chiral alcohols with high yields (74–95%) and 77–99% ee. We monitored the hydration/ATH sequential reactions by TLC for almost all the reactions and did not find any other by-products except for the incomplete transformation of the alkyne or intermediate acetophenone. Benefitting from the good tolerance of catalyst 2, the electronic properties and the structures of the substituents on the phenylacetylene derivatives did not significantly affect their enantioselectivity. In particular, the various electron-withdrawing and -donating substituents on the aromatic ring were equally efficient (83–95% yield and 92–99% ee, Entries 3–12) except for 4-nitrophenylacetylene which contained a strong electron-withdrawing group (74% yield and 77% ee, Entry 13). In addition to phenylacetylene derivatives, the 2-ethynylthiophene could also be converted to chiral heterocyclic alcohol with 91% yield and 97% ee (Table 1, entry 14) successfully. Furthermore, this one-pot hydration-ATH enantioselective sequential reaction could also be employed to synthesize chiral diols. As shown in Table 1, entry 15, the representative distereocentered diols were obtained, where the 1,4-diethynylbenzene catalyzed by hydration/ATH process led to corresponding chiral diols (83% yield and 99% *ee*) with high diastereoselectivity [90% diastereomeric excess (de)]. We also compared the activity of ATH reaction among the PMO@Ru in this work: SBA-15@Ru (Long, et al., 2013) and FDU-12@Ru (Gao, et al., 2013). The results indicate that both of them show high activity (up to 99% conv.) and enantioselectivity (up to 99% ee), but the catalyst loading (based on the content of Ru) for SBA-15@Ru and FDU-12@Ru was 1 mol% while PMO@Ru was 0.4 mol%. The activity and enantioselectivity could be maintained when catalyst loading of PMO@Ru was 0.2 mol% (entry 2, Supplementary Table S2). This result further confirms that the central-radical pore structure in the PMOs has a large pore size and highly accessible surface areas similar to the KCC-1 ((Fihri, et al., 2012). It not only acts as a storage reservoir for catalysts and guest molecules but also enables an efficient mass transfer in the hollow, thereby providing a promising platform for catalysis application.

**FIGURE 6:**
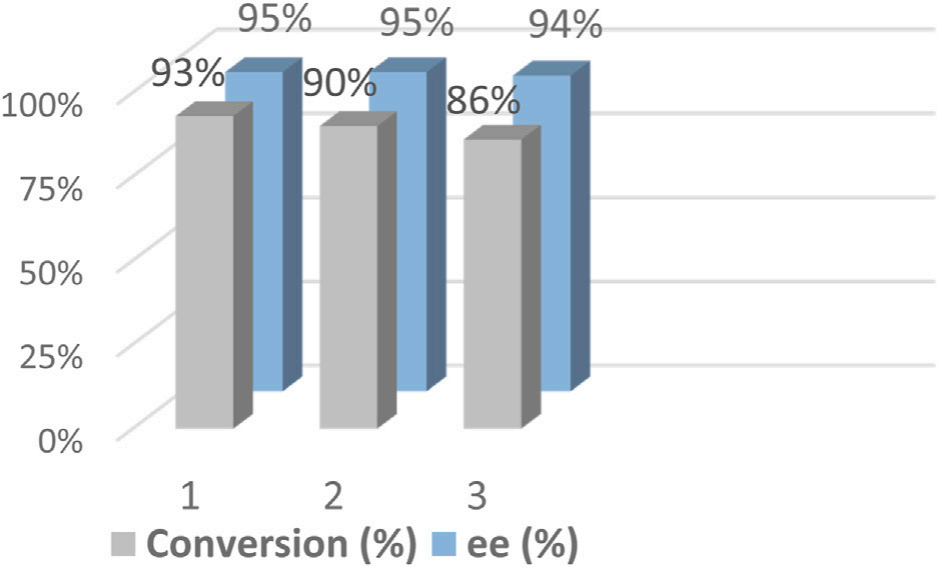
Reusability of catalyst 2 in the hydration–ATH of one-pot tandem reaction of phenylacetylene.

Another important purpose in the design of the heterogeneous DMONs-based catalyst 2 was the construction of a heterogeneous catalyst with high catalytic activity and high enantioselectivity for multiple cycles. We collected the solid catalyst 2 in the reaction mixture after completion of the reaction determined by TLC, and the precipitate was Soxhlet extracted with methanol and DCM until no catalyst 1 and product was detected in the eluent. The recovered solid was reactivated at 60°C under vacuum overnight and then reused for the next runs directly. The recycle studies were performed under half catalyst mass (1.0 mol% 1 and 0.2 mol% 2). The results show it afforded the target chiral alcohol with 86% conversion and 94% *ee* value at the third run (Figure 6, Supplementary Table S4B), indicating the activity of catalyst 2 decreased during the recycling. Then we conducted the recycling under the general condition (2.0 mol% 1 and 0.4 mol% 2) similar to the above substrates. The results for the one-pot sequential hydration-ATH reaction of phenylacetylene in the fifth consecutive reactions show that it afforded the target chiral alcohol with 92% conversion and 95% *ee* value (Supplementary Table S4A). The decreased conversion during the sixth recycle also happened. To figure out how that phenomenon happened, we studied the inductively coupled plasma (ICP) optical emission spectrometer analysis and found that the loss of Ru at the sixth recycle was 17.2% (4.351 mg/g), revealing a decreased amount of Ru in catalyst 2, corresponding to those of 6.9% (4.89 mg/g) at the fifth run, indicating that Ru leaching in 2 occurred. We also performed elemental analysis of the as-synthesized catalyst 2, and the recycled 2 after the sixth run. The results were as follow: *as-synthesized* ***2*** *: C 11.30, H 2.56, N 0.32, S 0.37* versus *Recycled* ***2*** *: C 11.09, H 2.52, N 0.21, S 0.37.* Compared to the N amounts in elemental analysis, it was found that the mole amount of N in the recycled 2 after the sixth run was 0.015 mmol per gram of 2 calculated from mass% of N atom (N 0.21%), meaning a loss of 0.0039 mmol of (DPEN) per gram of 2 (because per mole N atom is equivalent to about half mole amounts of DPEN atom). Meanwhile, ICP–OES analysis showed that the leaching of Ru was 0.0089 mmol (5.255–4.351 mg of Ru per gram of catalyst). Thus, the above results indicate that the low conversions at the sixth run may be caused by the lower content of the catalyst which originated from both the DPEN and Ru were lost during the recycling, especially since the decreased amount of Ru was larger than the DPEN. We, therefore, considered that the Ru leaching during the recycling was probably caused by the break of the sulfonamide bond or the coordination bond, especially because the weak alkaline conditions for the ATH reaction provide a suitable environment for hydrolysis. We also found that the amount of Co in 2 after the fifth recycle was 0.65 mg (0.0103 mmol) per gram in the ICP–OES analysis, indicating about 2.05% of Co was trapped in the pores of 2. We checked the hydration activity of the entrapped Co species, but none of the corresponding acetophenone was detected, indicating the inactivation of the entrapped Co species. The structural integrity of catalyst 2 was proven by solid-state ^13^C CP/MAS NMR and ^29^Si CP/MAS NMR spectra (Supplementary Figure S5 and Supplementary Figure S6). The catalytic activity was also confirmed by XPS analysis of 2 before and after sequential reaction (281.67 versus 281.79 eV, Supplementary Figure S7). The above results collectively illustrate that catalyst 2 was heterogeneous and a cyclable catalyst with slight catalyst leaching during the recycling.

## Conclusion

In conclusion, by combining the inexpensive cobaloximes with chiral Ru/diamine–functionality periodic mesoporous organosilica, we developed an economic and recyclable enantioselective cascade process for the facile synthesis of chiral alcohols through the control of hydration-ATH catalytic sequence. A variety of chiral alcohols were synthesized in good yield and up to 99% *ee* values. Additionally, the Ru/diamine complex immobilized onto the functionalized periodic mesoporous organosilica can be recycled in hydration-ATH one-pot sequential reaction of phenylacetylene more than three times in the case of Ru-leaching slowly. This work offers an operational approach to designing multifunctional heterogeneous co-catalysts for enantioselective sequential reactions.

## Data Availability

The original contributions presented in the study are included in the article/Supplementary Material, further inquiries can be directed to the corresponding authors.
