# Supplementary material for: Integrated Cobaloxime and Mesoporous Silica-Supported Ruthenium/Diamine Co-Catalysis for One-Pot Hydration/Reduction Enantioselective Sequential Reaction of Alkynes
Source: Front Chem. 2021 Sep 22;9:732542. doi: 10.3389/fchem.2021.732542 (PMC8493125; doi:10.3389/fchem.2021.732542)
Supplement: Supplementary file 1 [file DataSheet1.PDF]

Supplementary Material

Integrated Cobaloxime and Mesoporous Silica-supported Ruthenium/diamine Co-catalysts for One-pot Hydration/Reduction Enantioselective Sequential Reaction of Alkynes

Zeyang Liu, Yongjie Wang, Kaihong Liu, Shanshan Wang, Haocheng Liao, Yuanli Zhu, Baoming Hou, Chunxia Tan*, Guohua Liu*

CONTENTS

Experimental........ 2

Figure S1. FT-IR spectra 3

Figure S2. LC-MS spectra of catalyst **1**........ 4

Figure S3. UV-Vis spectra of catalyst **1** 5

Figure S4. NMR spectra of catalyst **1**........ 5

Figure S5-S6. Solid-state NMR spectra of catalyst 2 after recycle 6

Figure S7. XPS spectra........ 7

Table S1. **Control experiment for the one-pot hydration-ATH reaction** 8

Table S2. **Optimize the catalyst loading for the sequential reaction.** 8

Table S3. Reported results of related hydration and ATH reactions 9

Figure S8-S9. HPLC analysis........ 9

Table S4. Reusability of catalyst **2** 26

Figure S10. NMR spectra 25

References........ 34

Experimental

General. All reagents and solvents were purchased from commercial sources and used without further purification. Compound of Co(dmgBF$_2$)$_2$·2H$_2$O [*Chem. Commun.*, **2017**, *53*, 6926-6929.] and Compound of (*R*,*R*)-4- (trimethoxysilyl)ethyl)phenylsulfonyl-1,2-diphenylethylenediamine [*J. Mater. Chem.* **2010**, *20*, 1970-1975.] were synthesized according to the reported literatures.

Characterization.

The content of Ru in catalyst **2** was detected using an inductively coupled plasma optical emission spectrometer (ICP, Varian VISTA-MPX) after digested by aqua regia. Fourier transforms infrared (FT IR) spectra were collected on a Nicolet Magna 550 spectrometer using the KBr method. Scanning electron microscopy (SEM) images were obtained using a JEOL JSM-6380LV (WD 10 mm) microscope operating at 30 kV by FEM, and the powder sample is loaded on silicon wafer. the SEM results was analyzed by Hitachi S-4800 SEM FE PC-SEM. Transmission electron microscopy (TEM) images were performed on a JEOL JEM2010 electron microscope at an acceleration voltage of 220 kV, the light source is LbB$_6$ electron beam. And the image analyzed by digital micrograph. Nitrogen adsorption isotherms were measured at 77 K with a Quantachrome Nova 4000 analyzer. The samples were measured after being outgassed at 423 K overnight. Pore size distributions were calculated by using the BJH model (Desorption dV/dlog(D) Pore Volume, Halsey: Faas Correction, diameter range: 1.7 nm to 300.0 nm). The specific surface areas (SBET) of samples were determined from the linear parts of BET plots (p/p_0 = 0.1-0.3). Solid-state NMR experiments were explored on a Bruker AVANCE spectrometer at a magnetic field strength of 9.4 T with ^{1}H frequency of 400.1 MHz, ^{13}C frequency of 100.5 MHz, and ^{29}Si frequency of 79.4 MHz with 4 mm rotor at two spinning frequency of 5.5 kHz and 8.0 kHz, TPPM decoupling is applied in the during the acquisition period. ^{1}H cross-polarization in all-solid-state NMR experiments was employed using a contact time of 2 ms and the pulse lengths of 4 μs.

Figure S1. FT-IR spectra of catalyst **1**, DMONs, ArDPEN@DMONs and catalyst **2**

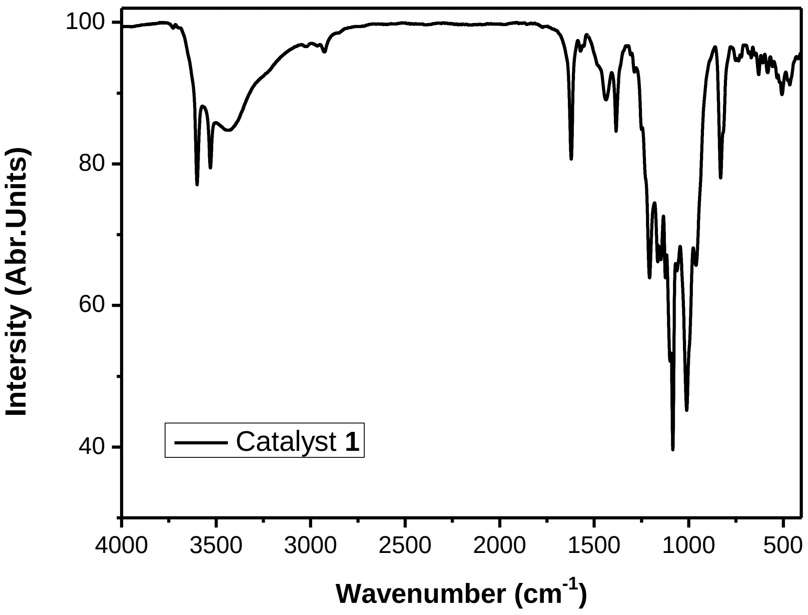

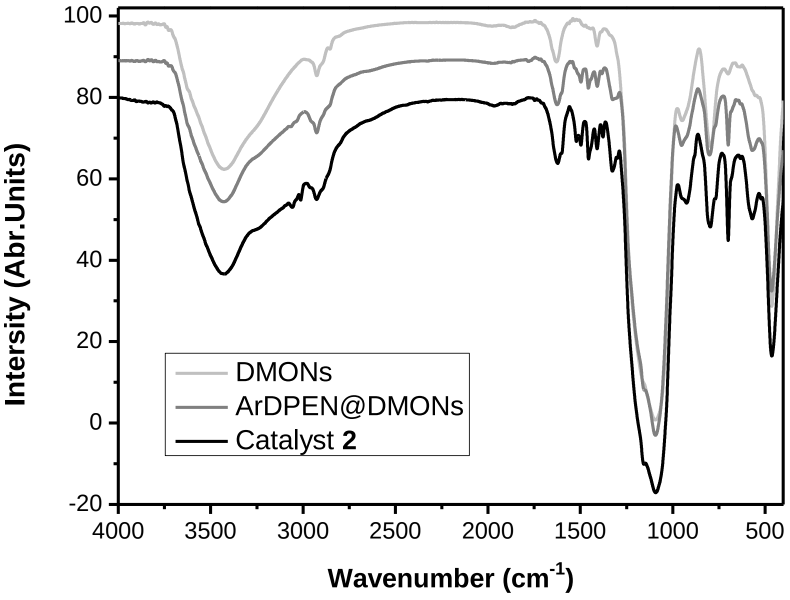

Figure S2 MS spectra of catalyst **1**

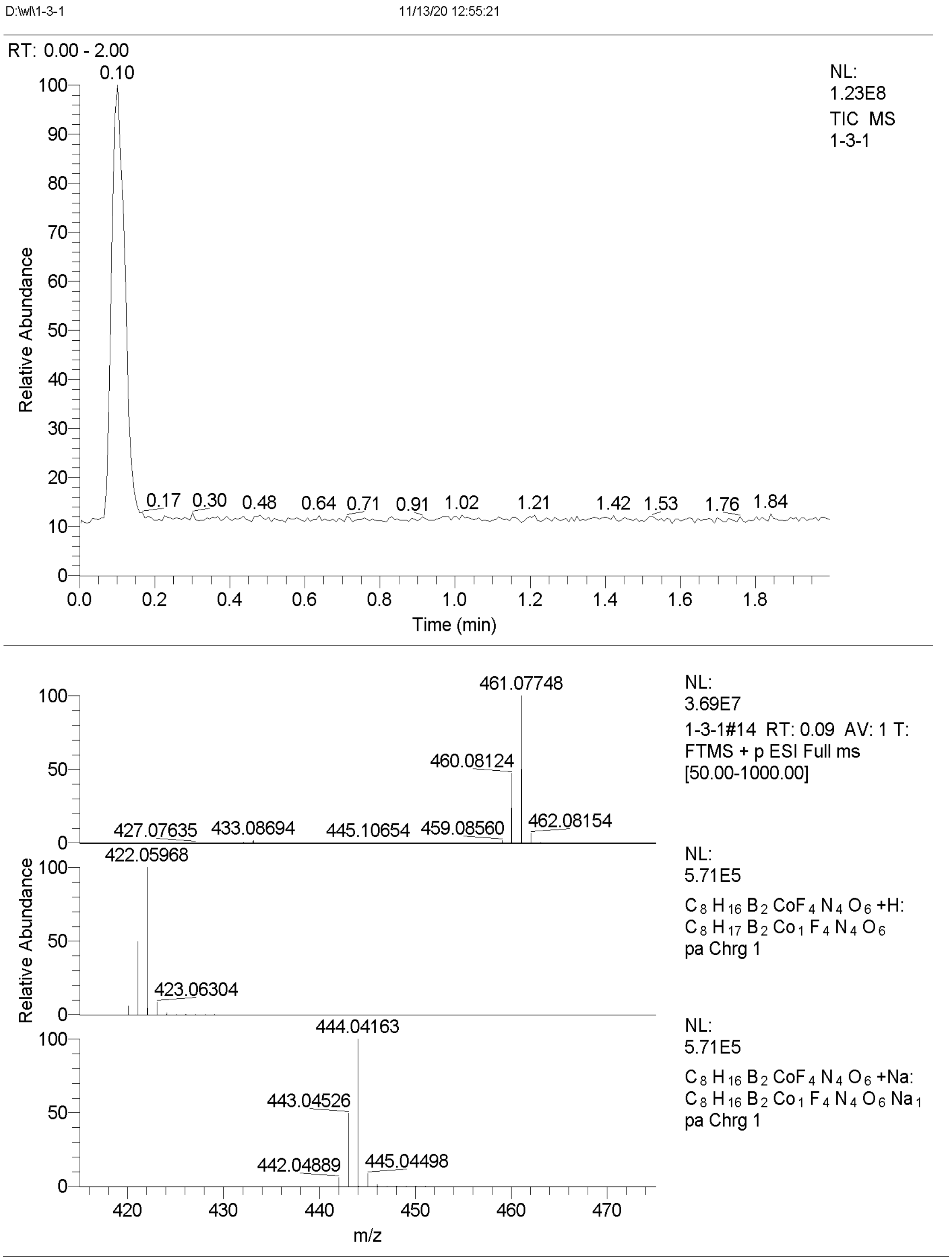

Figure S3 UV-Vis spectra of catalyst **1**

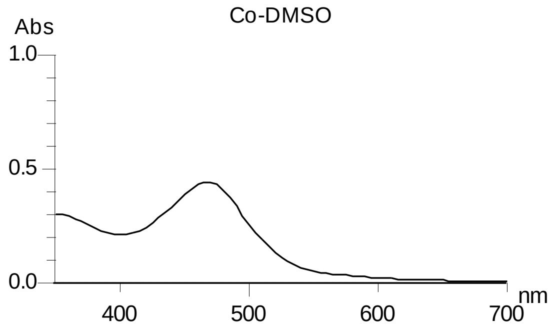

Figure S4 NMR spectra of catalyst **1**

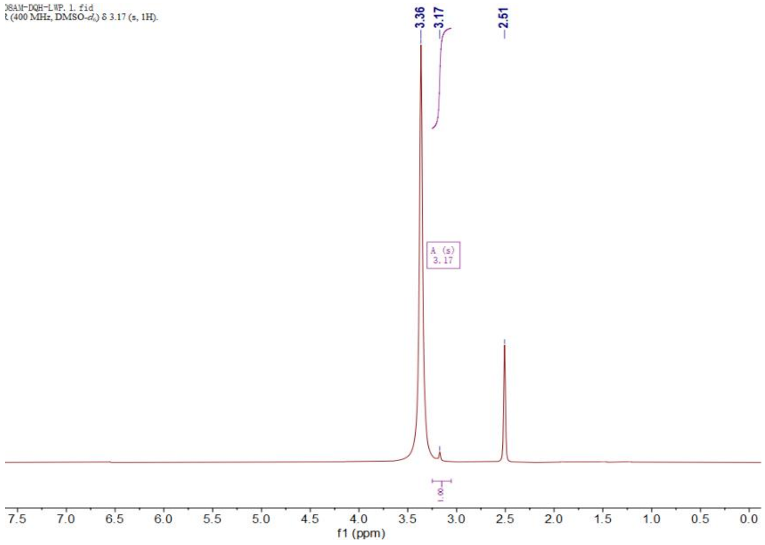

Figure S5 Solid-state ^{13}C CP/MAS NMR spectra of catalyst **2** after recycle

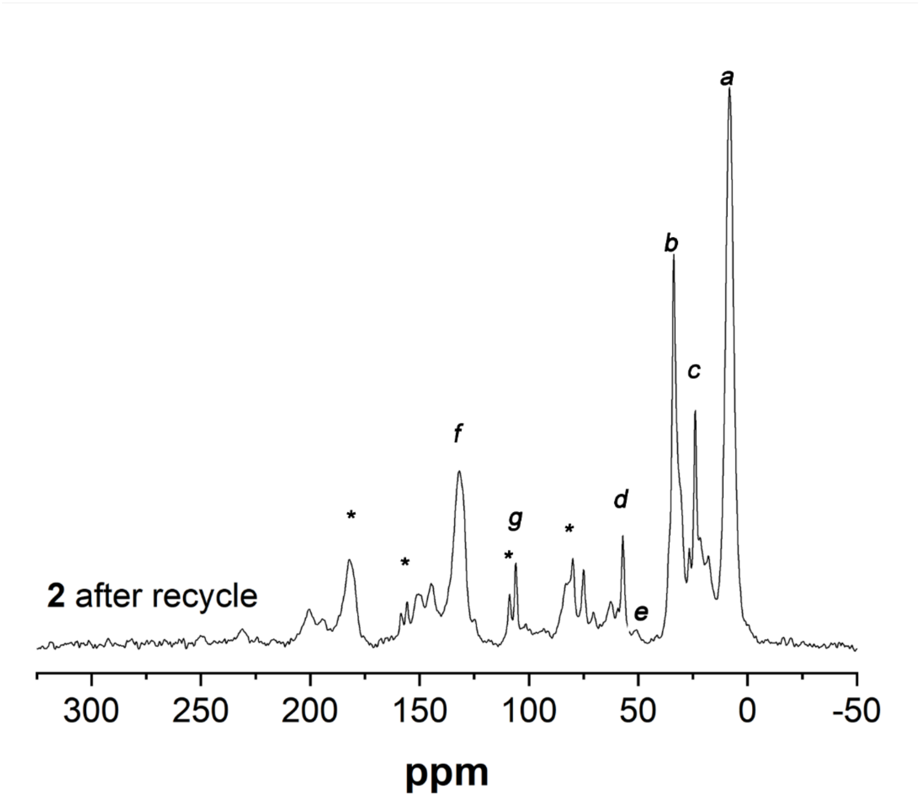

Figure S6 Solid-state ^{29}Si CP/MAS NMR spectra of catalyst **2** after recycle

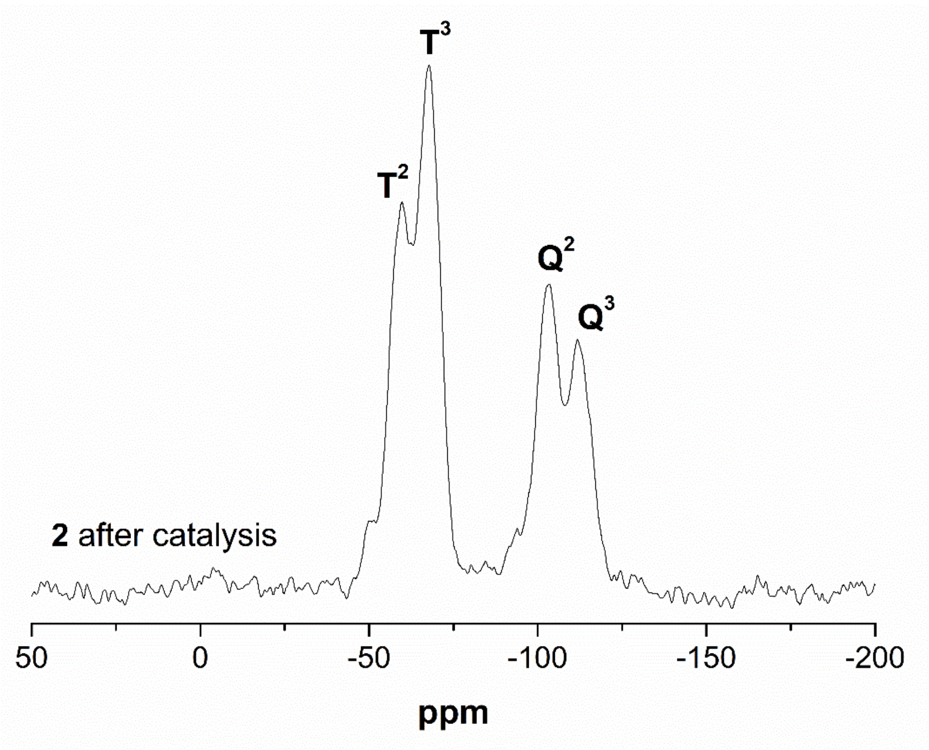

Figure S7 XPS spectra of the catalyst **2** before and after recycle

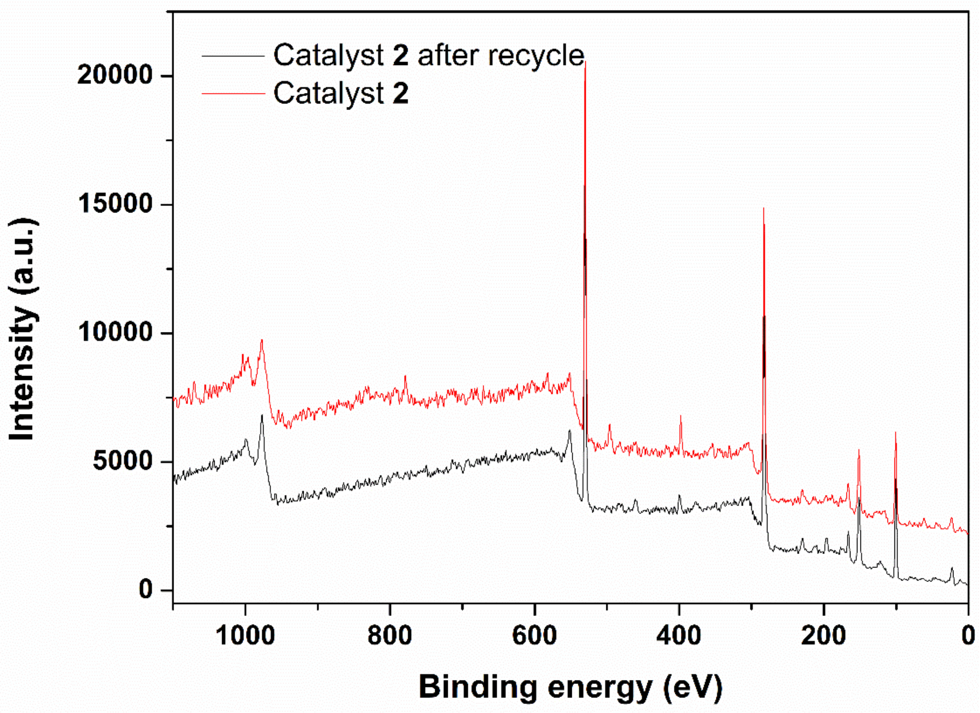

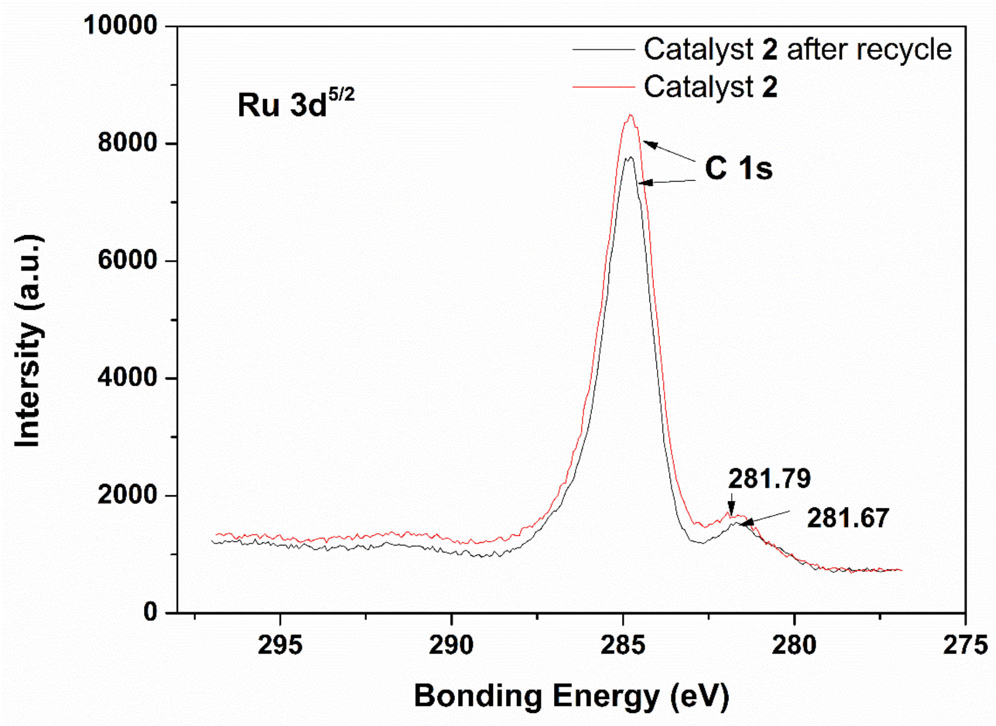

Table S1. Control experiment for the one-pot hydration-ATH reaction

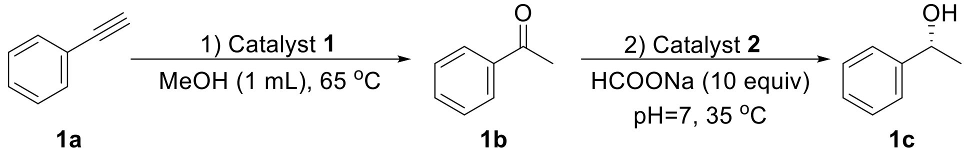

Entry	Cata. (%)	Substrate	Product	yield. (%)	ee[b] (%)
1	Catalyst **1**	**1a**	**1b**	99	-
2	Catalyst **1**[b][1]	**1a**	**ND**	-	-
3	Catalyst **2**	**1a**	ND	-	-
4	Catalyst **2**	**1b**	**1c**	97	96
5	Catalyst **2** [b]	**1b**	**1c**	96	95
6	Catalyst (**1**+**2**)[a]	**1a**	**1b**+**1c**	66+31	95
7	Catalyst (**1**+**2**)[b]	**1a**	ND	-	-
8	Catalyst (**1**+**2**)[b]	**1b**	**1c**	95	96
9	Catalyst (**1**+**2**)[c]	**1a**	**1c**	84	96

[a] Add the Catalyst **1** (2 mol%) and 2 (1 mol%) at the same time under aerobic conditions. [b] Add the Catalyst **1** (2 mol%) and **2** (1 mol%) at the same time under argon atmosphere.[c] The reaction performed via a step-wise method

Table S2. Optimize the catalyst loading for the sequential reaction.

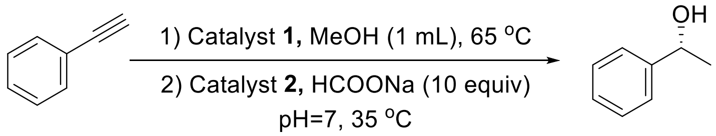

Entry	Cata. (%)	Loading (mol%)	Time (h)	yield. (%)	ee[b] (%)
1	Catalyst (**1**+**2**)[c]	**2 + 0.4**	5+12	84	96
2	Catalyst (**1**+**2**)[c]	**1 + 0.2**	5+12	85	96
3	Catalyst (**1**+**2**)[c]	**0.5 + 0.1**	8+18	80	95
4	Catalyst (**1**+**2**)[c]	**0.25 + 0.05**	12+24	67	59

[c] The reaction performed via a step-wise method

Table S3. Reported results of related hydration and ATH reactions.

Entry	catalyst	Condition (additive for hydration)	Yield	ee
1	[Rh][S1]	HCOOH, 100 °C	87	99
2	[(IPr)AuCl]+[Ru] [S2]	110 °C	97	97
3	[Salen-Co]+[Ru] [S3]	H_2SO_4, 80 °C	92	97
4	[Co-Porphyrin]+[Ru] [S4]	-	95	97
5	[TfOH]+[Rh] [S5]	20 atm (H_2)	96	99
6	[TfOH]+[Rh] [S6]		95	97

Figure S8. HPLC analyses for chiral products

(*R*)-1- Phenethylalcohol

Colorless oil; 84% yield, 96% ee, Purified by flash column chromatography (PE/EA 15/1). ^{1}H NMR (400 MHz, Chloroform-d) δ 7.34 – 7.25 (m, 4H), 7.25 – 7.18 (m, 1H), 4.77 (q, *J* = 6.5 Hz, 1H), 2.66 (s, 1H), 1.40 (d, *J* = 6.5 Hz, 3H). HPLC (OJ-H, elute: Hexanes/*i*-PrOH = 97/3, detector: 254 nm, flow rate: 1.0 mL/min, 25 °C, t_R = 16.92 min, t_S = 14.97 min).

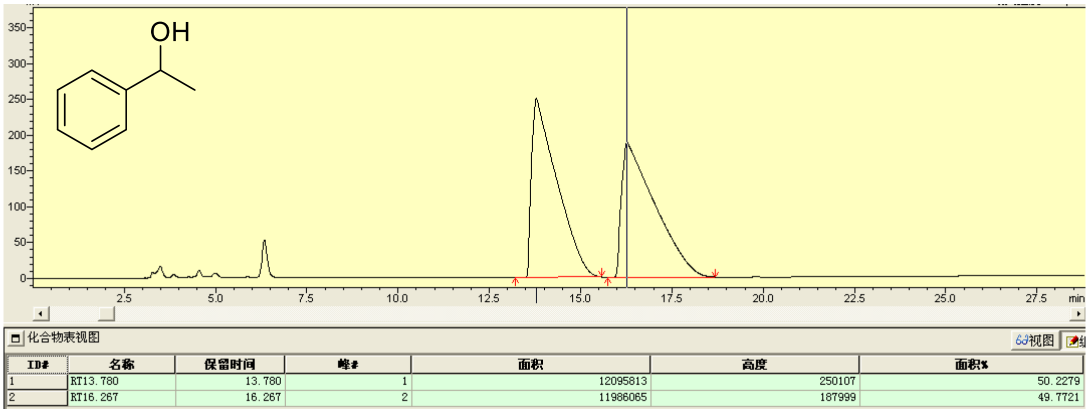

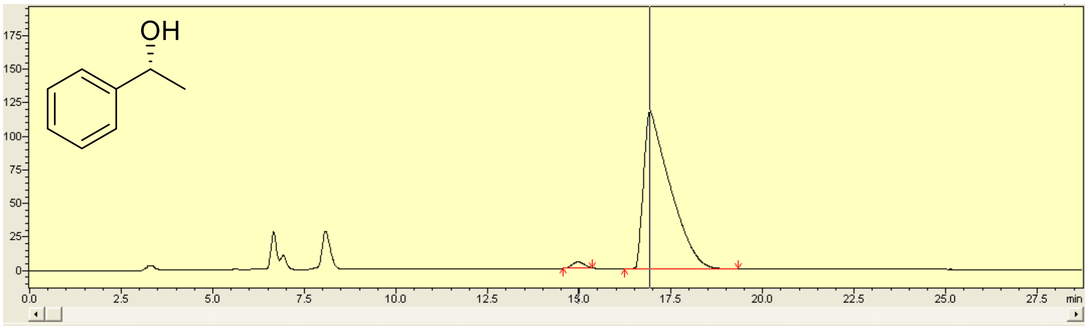

化合物表视图

ID#	名称	保留时间	峰#	面积	高度	面积%
1	RT14.968	14.968	1	107192	4611	1.7838
2	RT16.922	16.922	2	5901941	118295	98.2162

(*R*)-1-(p-tolyl)ethanol

Colorless oil; 85% yield, 96% ee, Purified by flash column chromatography (PE/EA 15/1). ^{1}H NMR (400 MHz, Chloroform-d) δ 7.20 (d, *J* = 8.2 Hz, 2H), 7.09 (d, *J* = 7.7 Hz, 2H), 4.80 (q, *J* = 6.4 Hz, 1H), 2.28 (s, 3H), 1.42 (d, *J* = 6.5 Hz, 3H). HPLC (OJ-H, elute: Hexanes/*i*-PrOH = 95/5, detector: 254 nm, flow rate: 1.0 mL/min, 25 ^{o}C, t_R = 11.09 min (major), t_S = 9.97 min (minor).

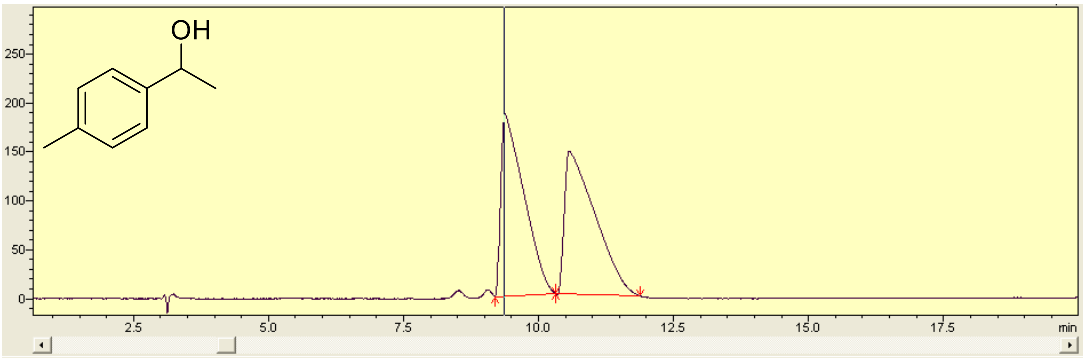

化合物表视图

ID#	名称	保留时间	峰#	面积	高度	面积%
1	RT9.375	9.375	1	5561899	186295	49.1462
2	RT10.569	10.569	2	5755152	146340	50.8538

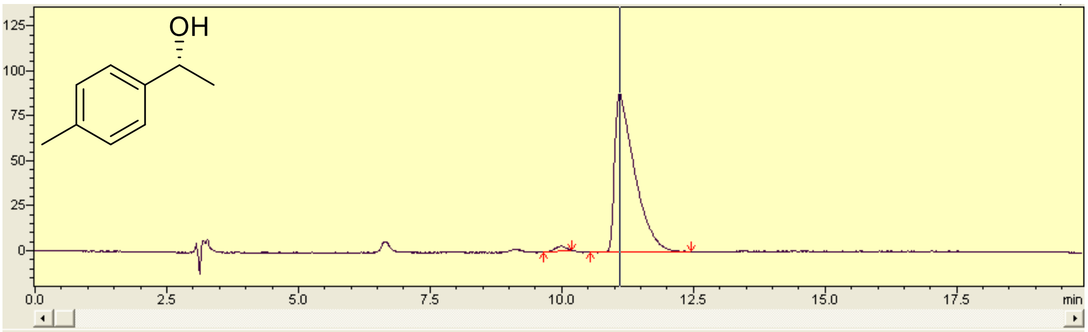

化合物表视图

ID#	名称	保留时间	峰#	面积	高度	面积%
1	RT9.978	9.978	1	40463	2872	1.7046
2	RT11.095	11.095	2	2333269	88678	98.2954

(*R*)-1-(4-ethylphenyl)ethanol

Colorless oil; 95% yield, 99% ee, Purified by flash column chromatography (PE/EA 15/1). ^{1}H NMR (400 MHz, Chloroform-d) δ 7.23 (d, *J* = 7.9 Hz, 2H), 7.12 (d, *J* = 7.8 Hz, 2H), 4.81 (q, *J* = 6.5 Hz, 1H), 2.58 (q, *J* = 7.6 Hz, 2H), 1.43 (d, *J* = 6.5 Hz, 3H), 1.17 (t, *J* = 7.6 Hz, 4H). HPLC (OD-H, elute: Hexanes/*i*-PrOH = 99/1, detector: 254 nm, flow rate: 1.0 mL/min, 25 ^{o}C, t_R = 19.20 min (major), t_S = 20.59 min (minor).

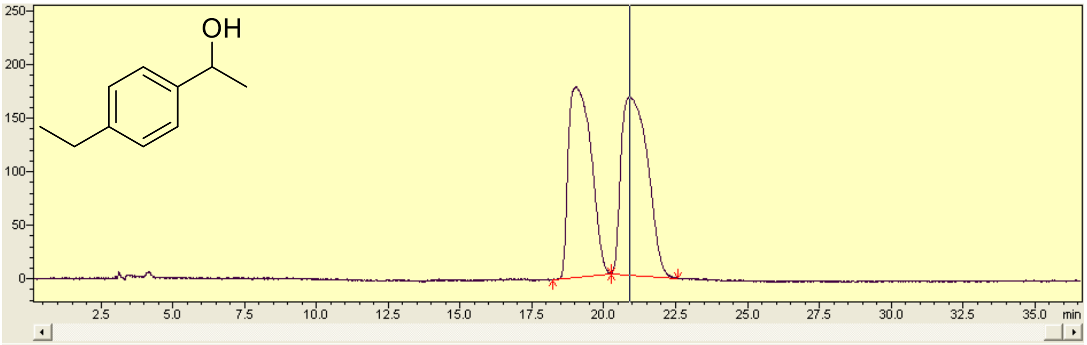

化合物表视图

ID#	名称	保留时间	峰#	面积	高度	面积%
1	RT19.042	19.042	1	9826739	177446	47.7371
2	RT20.882	20.882	2	10758366	166747	52.2629

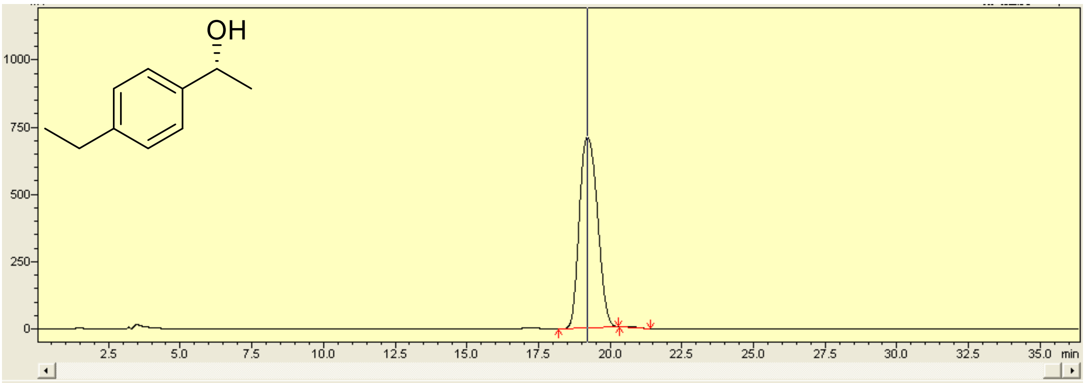

化合物表视图

ID#	名称	保留时间	峰#	面积	高度	面积%
1	RT19.205	19.205	1	31240512	709736	99.6913
2	RT20.596	20.596	2	96722	2284	0.3087

(*R*)-1-(4-propylphenyl)ethanol

Colorless oil; 93% yield, 99% ee, Purified by flash column chromatography (PE/EA 15/1). ^{1}H NMR (400 MHz, Chloroform-d) δ 7.23 (d, *J* = 7.9 Hz, 2H), 7.12 (d, *J* = 7.8 Hz, 2H), 4.81 (q, *J* = 6.5 Hz, 1H), 2.58 (q, *J* = 7.6 Hz, 2H), 1.43 (d, *J* = 6.5 Hz, 3H), 1.17 (t, *J* = 7.6 Hz, 4H). HPLC (AD-H, elute: Hexanes/*i*-PrOH = 98/2, detector: 215 nm, flow rate: 1.0 mL/min, 25 °C, t_R = 20.18 min (major), t_S = 23.59 min (minor).

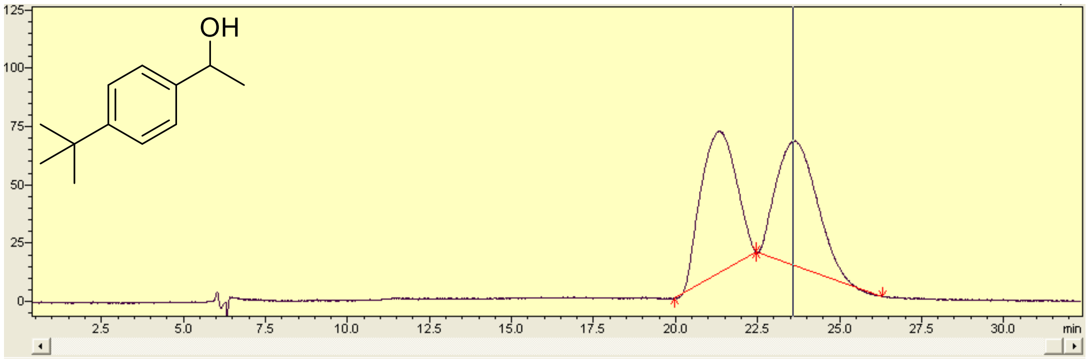

化合物表视图

ID#	名称	保留时间	峰#	面积	高度	面积%
1	RT21.318	21.318	1	4562772	61170	49.7728
2	RT23.591	23.591	2	4604421	53321	50.2272

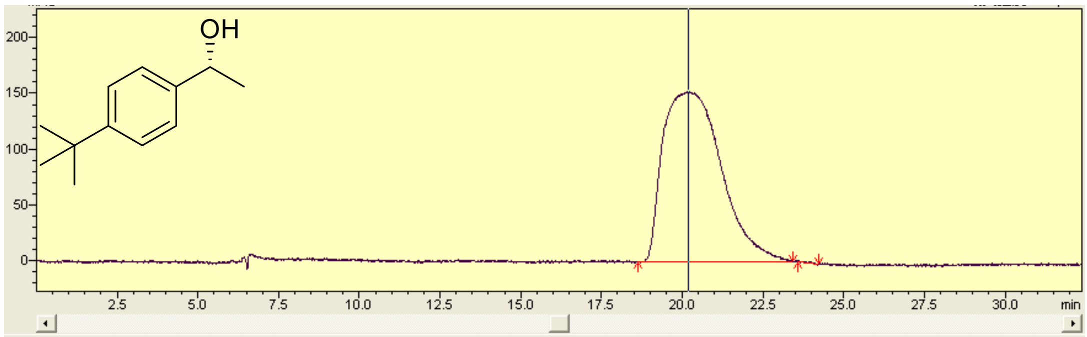

化合物表视图

ID#	名称	保留时间	峰#	面积	高度	面积%
1	RT20.185	20.185	1	19230296	153998	99.9608
2	RT23.592	23.592	2	7550	1383	0.0392

(*R*)-1-(4-methoxyphenyl)ethanol

Colorless oil; 84% yield, 97% ee, Purified by flash column chromatography (PE/EA 15/1). ^{1}H NMR (400 MHz, Chloroform-d) δ 7.23 – 7.16 (m, 2H), 6.91 – 6.85 (m, 2H), 4.81 (q, *J* = 6.5 Hz, 1H), 3.75 (s, 3H), 1.42 (d, *J* = 6.5 Hz, 3H). HPLC (OD-H, elute: Hexanes/*i*-PrOH = 97/3, detector: 254 nm, flow rate: 1.0 mL/min, 25 ^{o}C, t_R = 23.13 min (major), t_S = 16.52 min (minor).

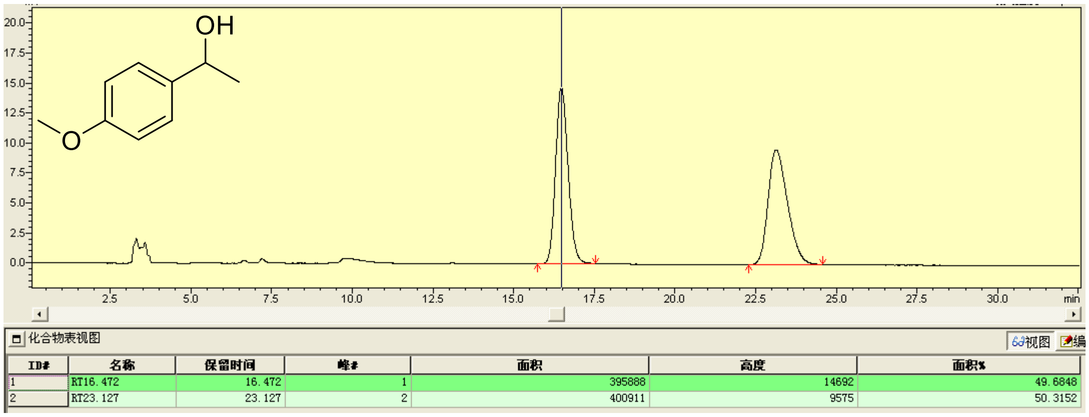

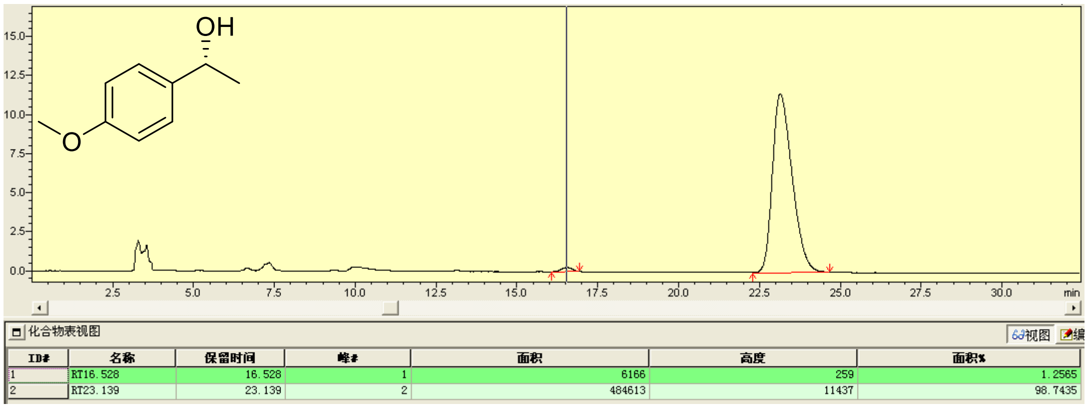

(*R*)-1-(2-methoxyphenyl)ethanol

Colorless oil; 89% yield, 99% ee, Purified by flash column chromatography (PE/EA 15/1). ^{1}H NMR (400 MHz, Chloroform-d) δ 7.37 (dd, *J* = 7.5, 1.7 Hz, 1H), 7.32 – 7.24 (m, 1H), 6.99 (td, *J* = 7.5, 1.1 Hz, 1H), 6.91 (dd, *J* = 8.2, 1.1 Hz, 1H), 5.12 (q, *J* = 6.5 Hz, 1H), 3.89 (s, 3H), 1.53 (d, *J* = 6.5 Hz, 3H). HPLC (OJ-H, elute: Hexanes/*i*-PrOH = 97/3, detector: 254 nm, flow rate: 1.0 mL/min, 25 °C, t_R = 16.34 min (major), t_S = 19.50 min (minor).

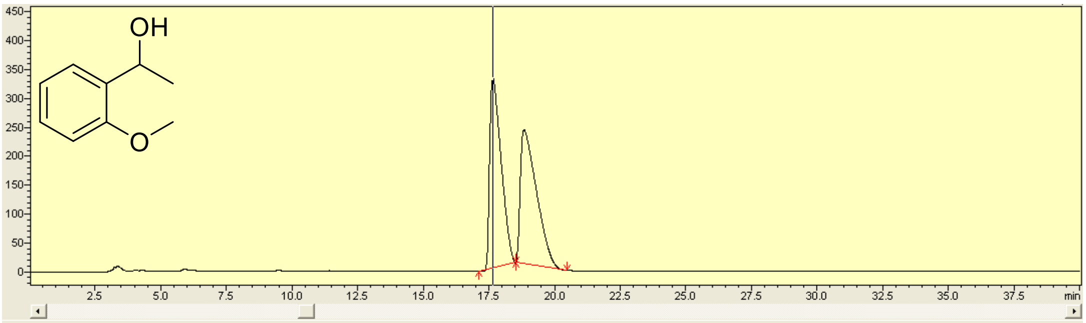

化合物表视图

ID#	名称	保留时间	峰#	面积	高度	面积%
1	RT17.649	17.649	1	9986820	329355	49.9889
2	RT18.839	18.839	2	9991267	232667	50.0111

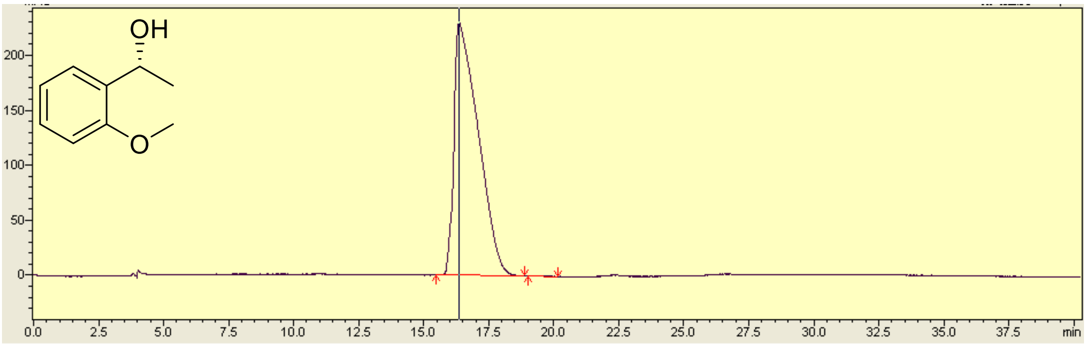

化合物表视图

ID#	名称	保留时间	峰#	面积	高度	面积%
1	RT16.348	16.348	1	14865419	230111	99.9496
2	RT19.506	19.506	2	7502	379	0.0504

(*R*)-1-(3-methoxyphenyl)ethanol

Colorless oil; 89% yield, 93% ee, Purified by flash column chromatography (PE/EA 15/1). ^{1}H NMR (400 MHz, Chloroform-d) δ 7.31 – 7.27 (m, 1H), 7.01 – 6.91 (m, 2H), 6.83 (ddd, *J* = 8.2, 2.5, 1.1 Hz, 1H), 4.89 (q, *J* = 6.5 Hz, 1H), 3.84 (s, 3H), 1.51 (d, *J* = 6.5 Hz, 3H). HPLC (OJ-H, elute: Hexanes/*i*-PrOH = 97/3, detector: 254 nm, flow rate: 1.0 mL/min, 25 °C, t_R = 28.05 min (major), t_S = 25.63 min (minor).

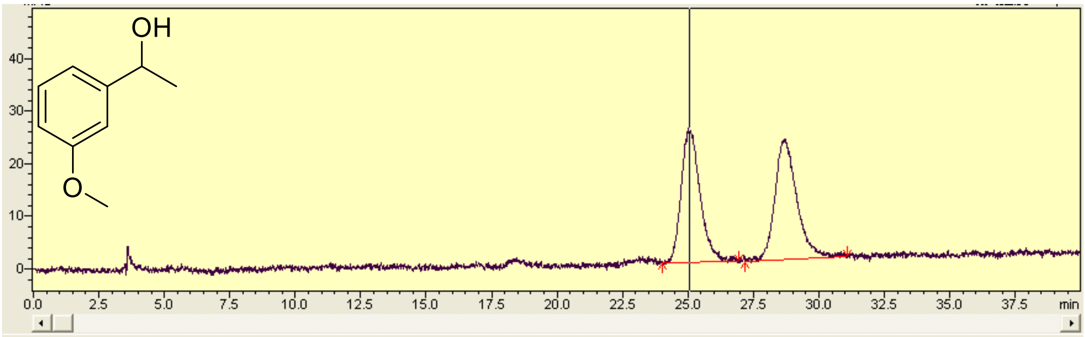

化合物表视图

ID#	名称	保留时间	峰#	面积	高度	面积%
1	RT25.007	25.007	1	1265712	25491	48.9518
2	RT28.679	28.679	2	1319915	22785	51.0482

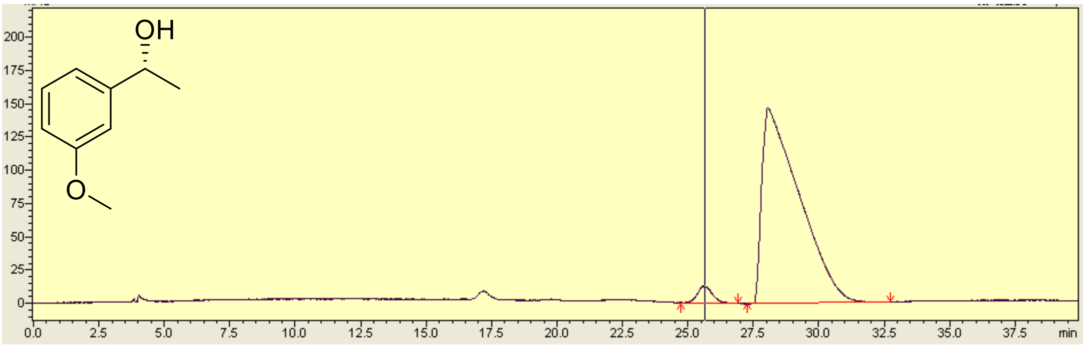

化合物表视图

ID#	名称	保留时间	峰#	面积	高度	面积%
1	RT25.635	25.635	1	497149	12791	3.2835
2	RT28.058	28.058	2	14643798	147209	96.7165

(*R*)-1-(4-fluorophenyl)ethanol

Colorless oil; 93% yield, 95% ee, Purified by flash column chromatography (PE/EA 15/1). ^{1}H NMR (400 MHz, Chloroform-d) δ 7.27 (ddq, J = 8.5, 5.1, 3.0 Hz, 2H), 7.03 – 6.88 (m, 2H), 4.82 (q, J = 6.5 Hz, 1H), 1.41 (d, J = 6.3 Hz, 3H). HPLC (AS-H, elute: Hexanes/*i*-PrOH = 93/7, detector: 254 nm, flow rate: 1.0 mL/min, 25 °C, t_R = 7.07 min (major), t_S = 6.51 min (minor).

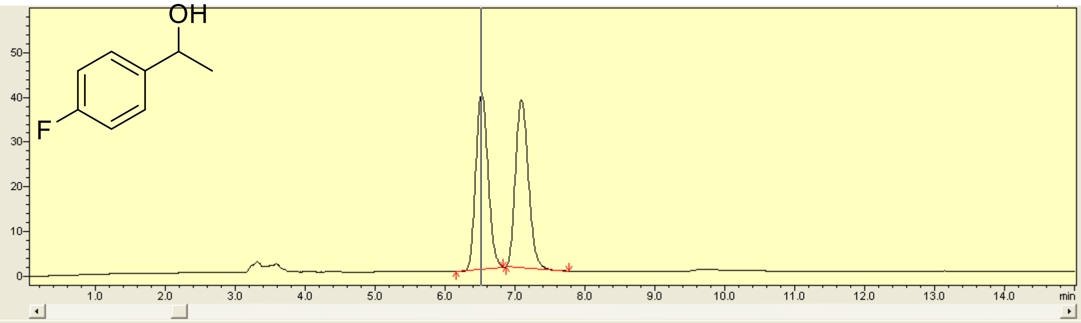

化合物表视图

ID#	名称	保留时间	峰#	面积	高度	面积%
1	RT6.518	6.518	1	479704	39477	49.9884
2	RT7.088	7.088	2	479927	37552	50.0116

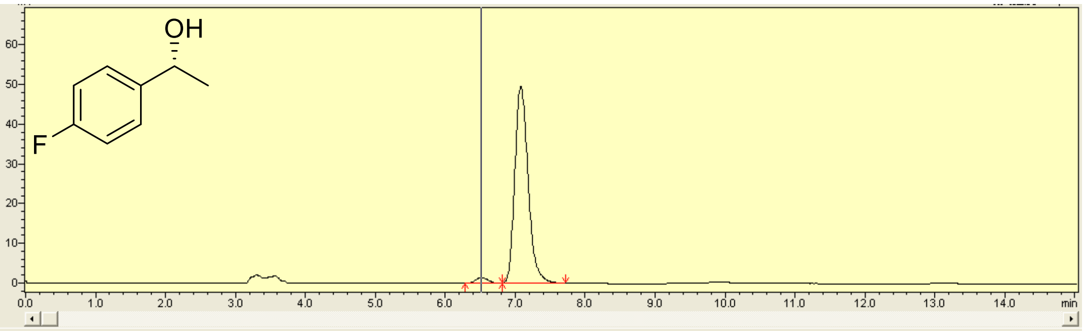

化合物表视图

ID#	名称	保留时间	峰#	面积	高度	面积%
1	RT6.514	6.514	1	17181	1407	2.5442
2	RT7.076	7.076	2	658104	49620	97.4558

(*R*)-1-(4-bromophenyl)ethanol

Colorless oil; 91% yield, 92% ee, Purified by flash column chromatography (PE/EA 15/1). ^{1}H NMR (400 MHz, Chloroform-d) δ 7.40 (d, *J* = 8.5 Hz, 2H), 7.18 (d, *J* = 8.3 Hz, 2H), 4.80 (q, *J* = 6.4 Hz, 1H), 1.40 (d, *J* = 6.7 Hz, 3H). HPLC (OD-H, elute: Hexanes/*i*-PrOH = 95/5, detector: 254 nm, flow rate: 1.0 mL/min, 25 ^{o}C, t_R = 8.90 min (major), t_S = 8.14 min (minor).

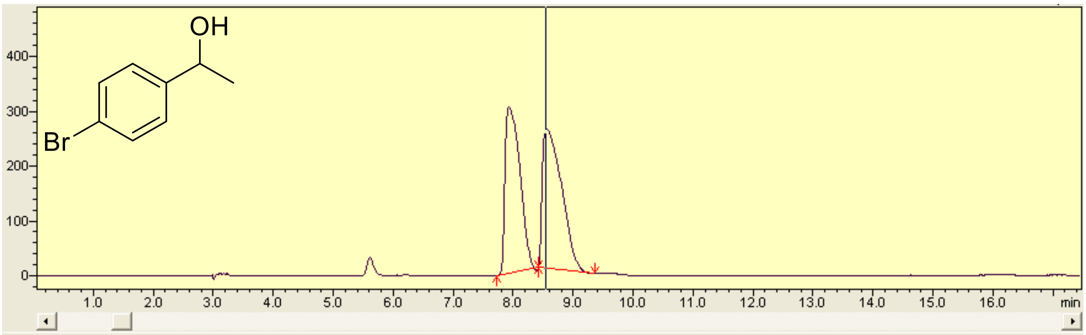

化合物表视图

ID#	名称	保留时间	峰#	面积	高度	面积%
1	RT7.928	7.928	1	5204758	302609	47.9789
2	RT8.553	8.553	2	5643256	253291	52.0211

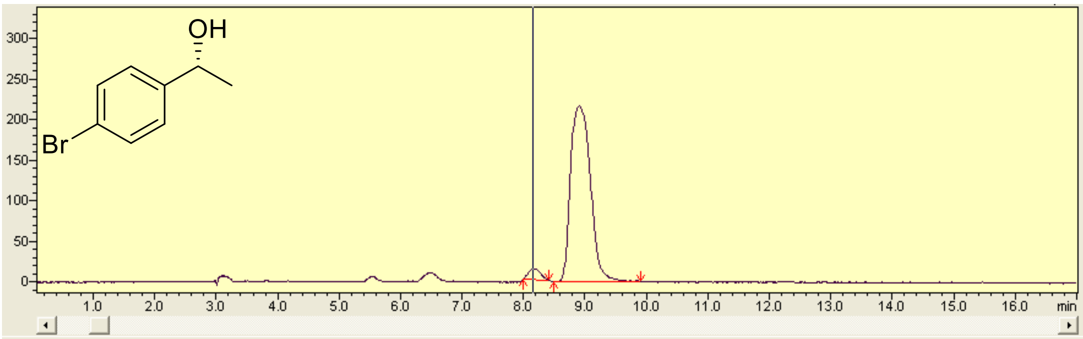

化合物表视图

ID#	名称	保留时间	峰#	面积	高度	面积%
1	RT8.148	8.148	1	185344	13690	3.6710
2	RT8.909	8.909	2	4863571	216382	96.3290

(*R*)-1-(4-chlorophenyl)ethanol

Colorless oil; 93% yield, 93% ee, Purified by flash column chromatography (PE/EA 15/1). ^{1}H NMR (400 MHz, Chloroform-d) δ 7.24 (s, 2H), 4.88 – 4.75 (m, 1H), 1.40 (dd, J = 6.4, 1.2 Hz, 3H). HPLC (OD-H, elute: Hexanes/*i*-PrOH = 97/3, detector: 254 nm, flow rate: 1.0 mL/min, 25 °C, t_R = 11.97 min (major), t_S = 10.84 min (minor).

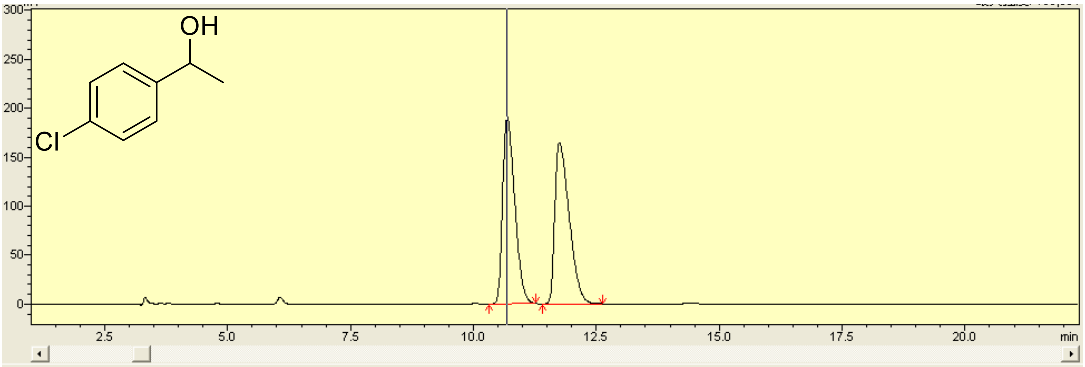

化合物表视图

ID#	名称	保留时间	峰#	面积	高度	面积%
1	RT10.685	10.685	1	3276985	190809	49.3744
2	RT11.750	11.750	2	3360021	164936	50.6256

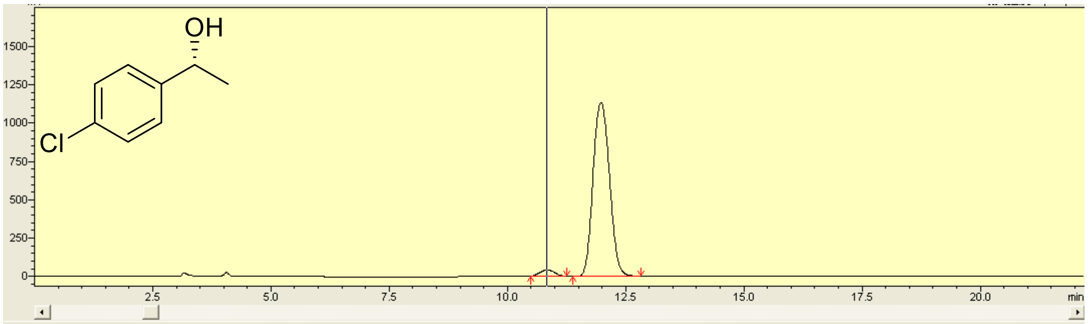

化合物表视图

ID#	名称	保留时间	峰#	面积	高度	面积%
1	RT10.840	10.840	1	904813	41143	3.1937
2	RT11.974	11.974	2	27426271	1131051	96.8063

(*R*)-1-(4-nitrophenyl)ethanol

Brown oil; 74% yield, 77% ee, Purified by flash column chromatography (PE/EA 8/1). ^{1}H NMR (400 MHz, Chloroform-d) δ 8.23 (d, J = 8.8 Hz, 2H), 7.57 (d, J = 8.2 Hz, 2H), 5.05 (q, J = 6.5 Hz, 1H), 1.55 (d, J = 6.5 Hz, 3H). HPLC (OJ-H, elute: Hexanes/*i*-PrOH = 95/5, detector: 254 nm, flow rate: 1.0 mL/min, 25 °C, t_R = 34.48 min (major), t_S = 31.71 min (minor).

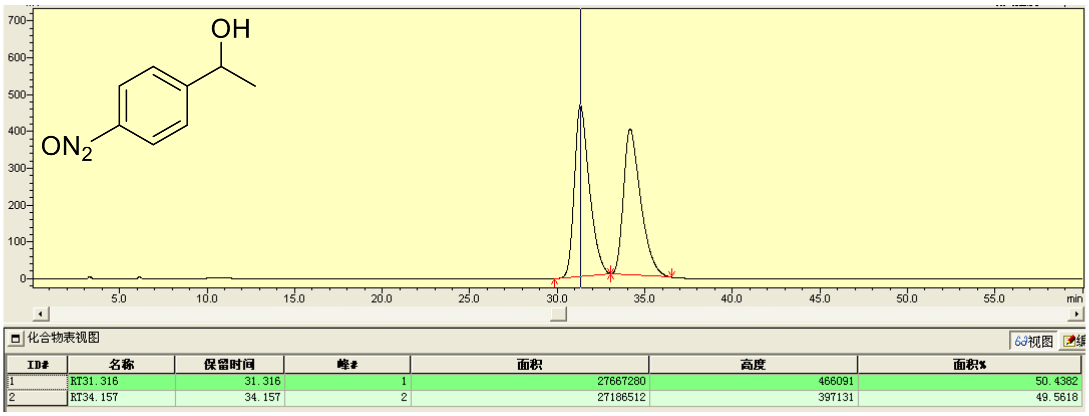

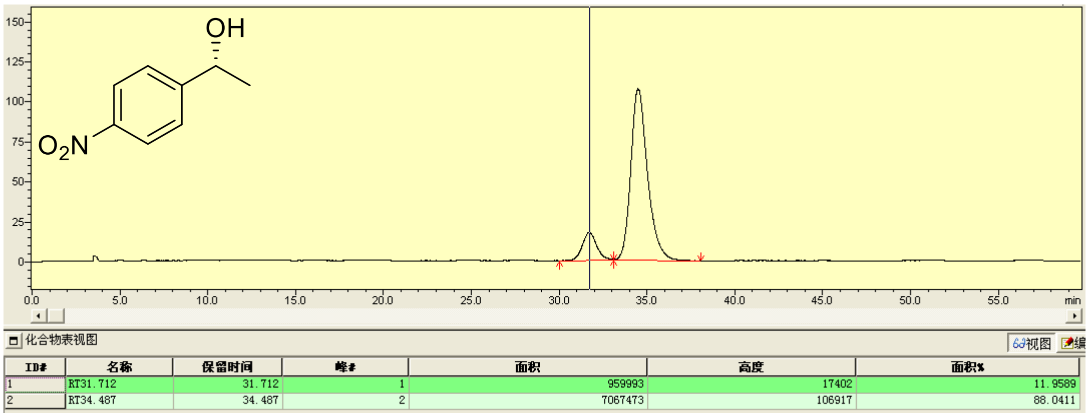

(*R*)-1-(3-bromophenyl)ethanol

Colorless oil; 93% yield, 99% ee, Purified by flash column chromatography (PE/EA 15/1). ^{1}H NMR (400 MHz, Chloroform-d) δ 7.56 (t, *J* = 1.9 Hz, 1H), 7.42 (dt, *J* = 7.8, 1.6 Hz, 1H), 7.32 (d, *J* = 7.7 Hz, 1H), 7.24 (t, *J* = 7.8 Hz, 1H), 4.90 (q, *J* = 6.4 Hz, 1H), 1.51 (d, *J* = 6.5 Hz, 3H). HPLC (OD-H, elute: Hexanes/*i*-PrOH = 95/5, detector: 220 nm, flow rate: 1.0 mL/min, 25 °C, t_R = 7.98 min (major), t_S = 8.94 min (minor).

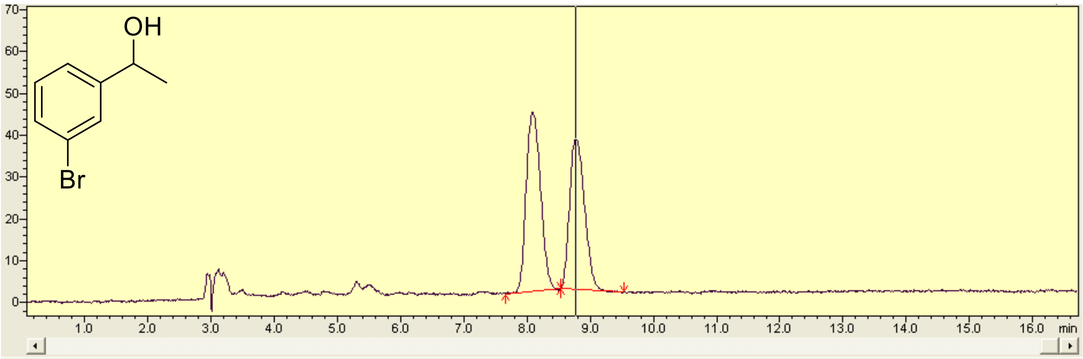

化合物表视图

ID#	名称	保留时间	峰#	面积	高度	面积%
1	RT8.094	8.084	1	674038	42864	53.2971
2	RT8.735	8.771	2	590642	36095	46.7029

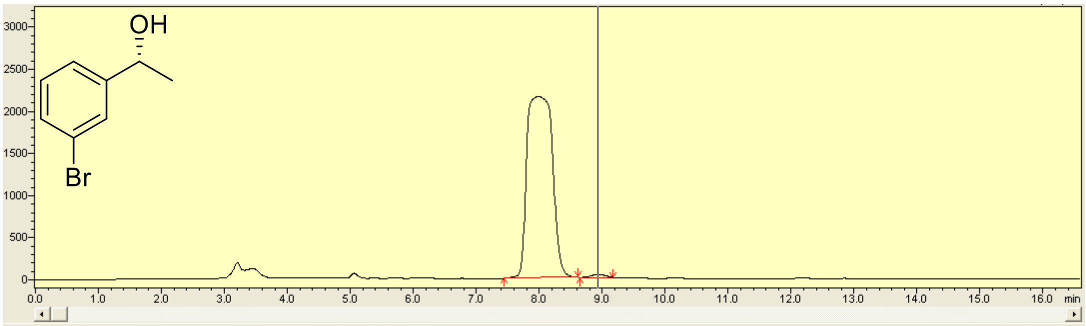

化合物表视图

ID#	名称	保留时间	峰#	面积	高度	面积%
1	RT7.984	7.984	1	60371403	2151525	99.1163
2	RT8.940	8.940	2	538231	34946	0.8837

(*R*) -1-(2-Thiophen)ethanol

Colorless oil; 91% yield, 97% ee, Purified by flash column chromatography (PE/EA 10/1). ^{1}H NMR (400 MHz, Chloroform-d) δ 7.27 (dd, *J* = 4.8, 1.4 Hz, 1H), 7.03 – 6.97 (m, 2H), 5.17 (q, *J* = 6.4 Hz, 1H), 1.63 (d, *J* = 6.4 Hz, 3H). HPLC (OJ-H, elute: Hexanes/i-PrOH = 95/5, detector: 254 nm, flow rate: 1.0 mL/min, 25 oC, t_R = 15.59 min (major), t_S = 12.24 min (minor).

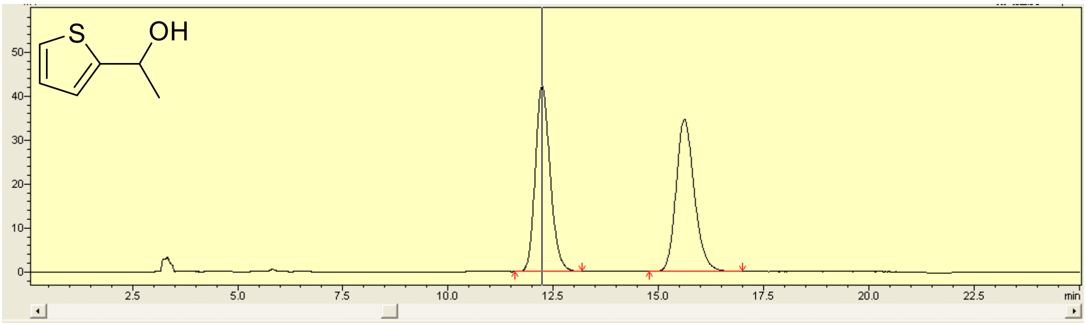

化合物表视图

ID#	名称	保留时间	峰#	面积	高度	面积%
1	RT12. 227	12. 227	1	1038154	42274	49. 7598
2	RT15. 607	15. 607	2	1048178	34715	50. 2402

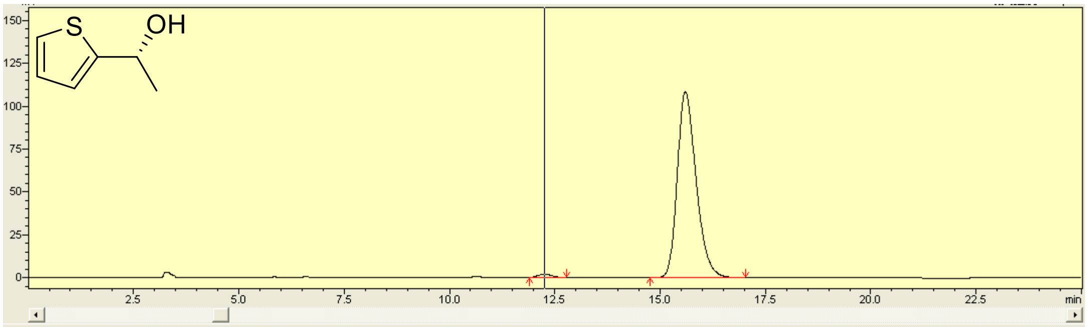

化合物表视图

ID#	名称	保留时间	峰#	面积	高度	面积%
1	RT12. 247	12. 247	1	43979	1919	1. 2885
2	RT15. 596	15. 596	2	3369308	108252	98. 7115

(1*R*,1'*R*)-1,1'-(1,4-phenylene)diethanol

Colorless oil; 83% yield, 99% ee 9:1 dr, Purified by flash column chromatography (PE/EA 15/1). ^{1}H NMR (400 MHz, Chloroform-d) δ 7.39 (s, 4H), 4.93 (q, J = 6.4 Hz, 2H), 1.52 (d, J = 6.4 Hz, 6H). HPLC (OD-H, elute: Hexanes/i-PrOH = 90/10, detector: 215 nm, flow rate: 1.0 mL/min, 25 ^{o}C, $t_{R,\,R}$ = 12.31 min (major), $t_{S,\,S}$ = 13.69 min (minor), $t_{R,\,S}$ = 18.28 min.

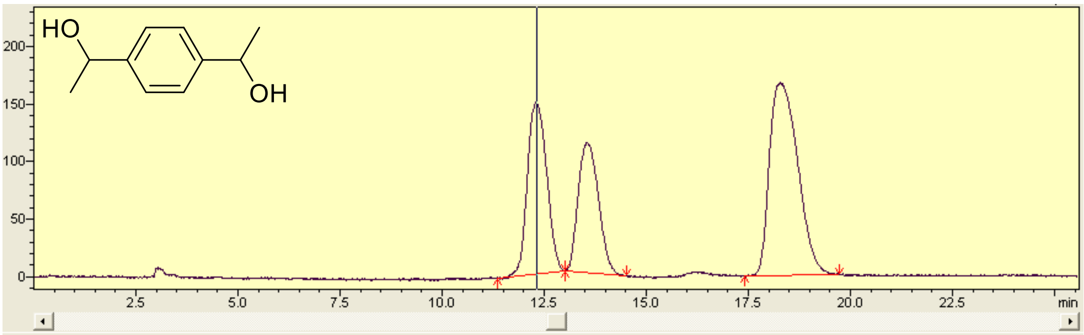

化合物表视图

ID#	名称	保留时间	峰#	面积	高度	面积%
1	RT12.320	12.307	1	4912623	149621	29.0216
2	RT13.565	13.549	2	3907983	113376	23.0867
3	RT18.287	18.288	3	8106852	168325	47.8917

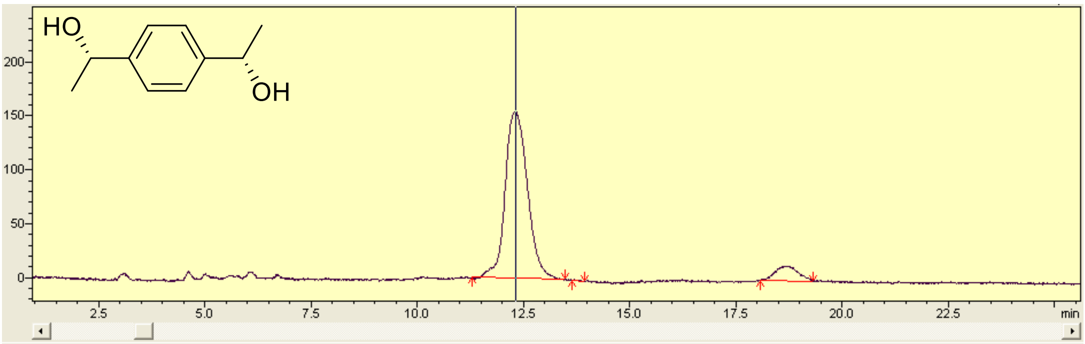

化合物表视图

ID#	名称	保留时间	峰#	面积	高度	面积%
1	RT12.310	12.310	1	5606717	154593	91.8632
2	RT13.693	13.693	2	2870	662	0.0470
3	RT18.671	18.671	3	493746	13213	8.0898

(*R*)-1- Phenethylalcohol[e, f, g]

Adjust pH =3[e], 12[f], 10[g]

pH=3

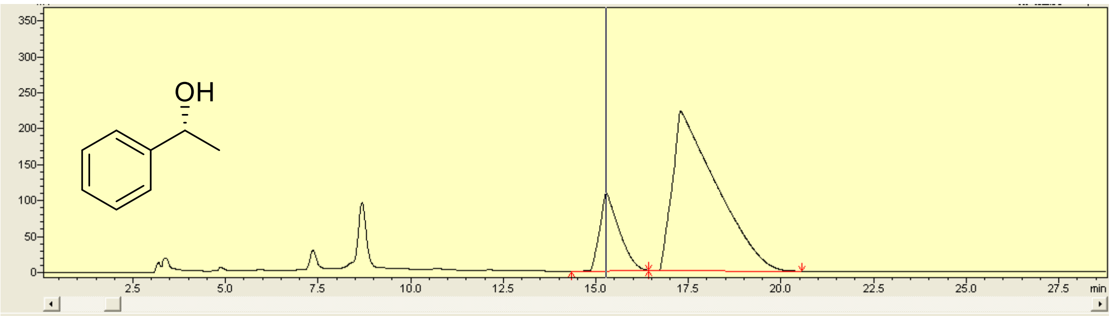

化合物表视图

ID#	名称	保留时间	峰#	面积	高度	面积%
1	RT15. 277	15. 277	1	3900759	107815	17. 5596
2	RT17. 287	17. 287	2	18313683	221665	82. 4404

pH=10

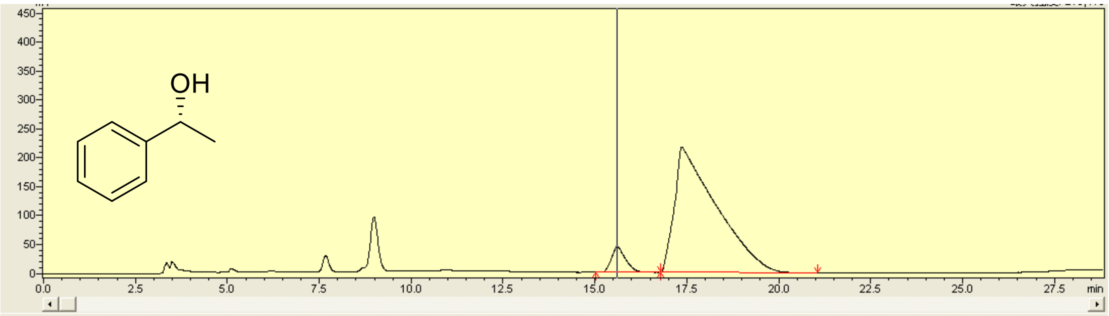

化合物表视图

ID#	名称	保留时间	峰#	面积	高度	面积%
1	RT15. 601	15. 601	1	1165595	44404	6. 3601
2	RT17. 359	17. 359	2	17161128	216507	93. 6399

pH=12

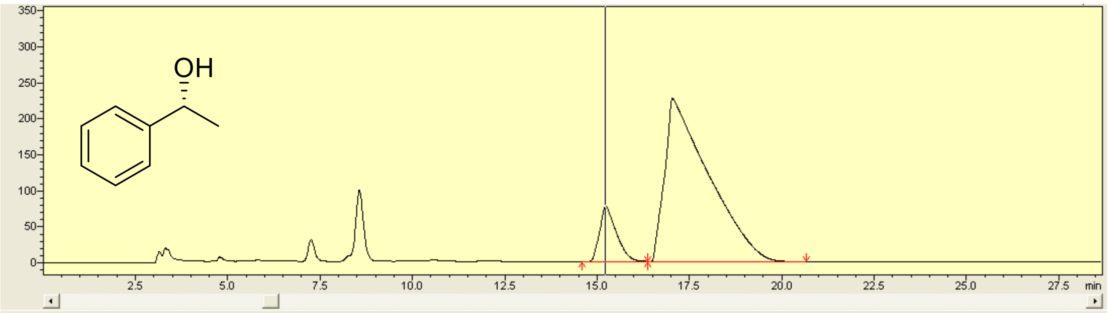

化合物表视图

ID#	名称	保留时间	峰#	面积	高度	面积%
1	RT15. 231	15. 231	1	2391470	77299	11. 3562
2	RT17. 042	17. 042	2	18667224	226132	88. 6438

Figure S9. Reusability of catalyst **2**

Racmic

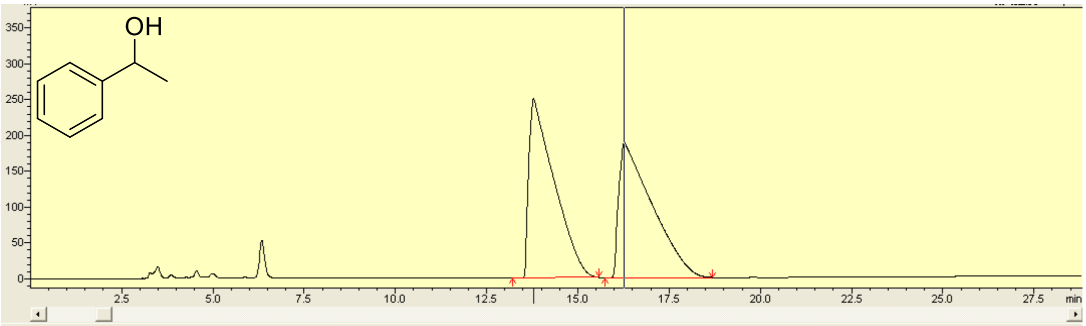

化合物表视图

ID#	名称	保留时间	峰#	面积	高度	面积%
1	RT13. 780	13. 780	1	12095813	250107	50. 2279
2	RT16. 267	16. 267	2	11986065	187999	49. 7721

Run 1

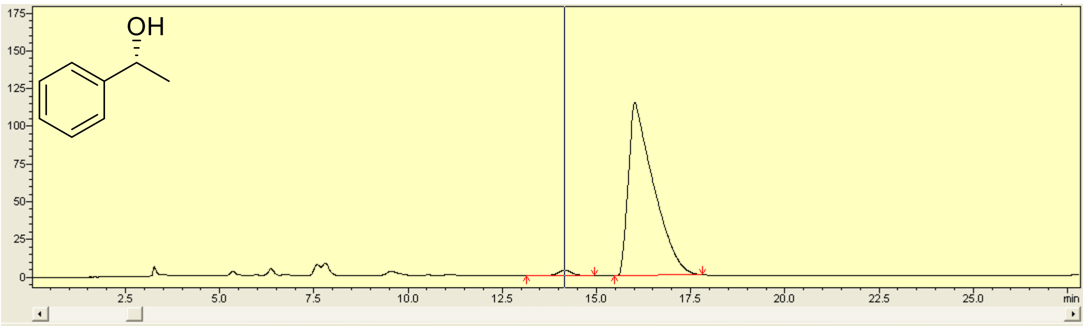

化合物表视图

ID#	名称	保留时间	峰#	面积	高度	面积%
1	RT14. 161	14. 161	1	85811	3521	1. 5828
2	RT16. 014	16. 014	2	5335675	114842	98. 4172

Run 2

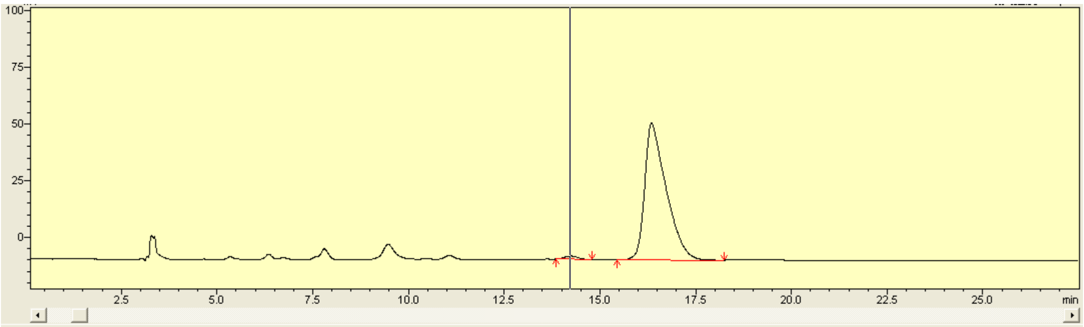

化合物表视图

ID#	名称	保留时间	峰#	面积	高度	面积%
1	RT14. 227	14. 227	1	36620	1468	1. 5329
2	RT16. 343	16. 343	2	2352396	60473	98. 4671

Run 3

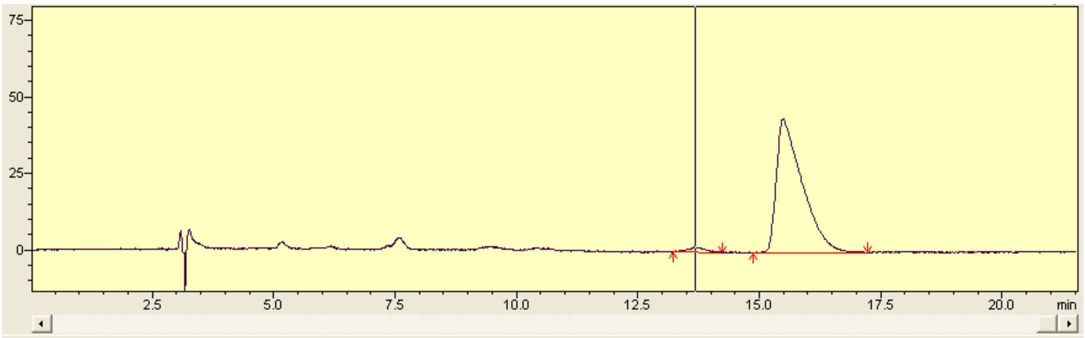

化合物表视图

ID#	名称	保留时间	峰#	面积	高度	面积%
1	RT13.678	13.678	1	35927	1564	2.1697
2	RT15.490	15.490	2	1619932	43623	97.8303

Run 4

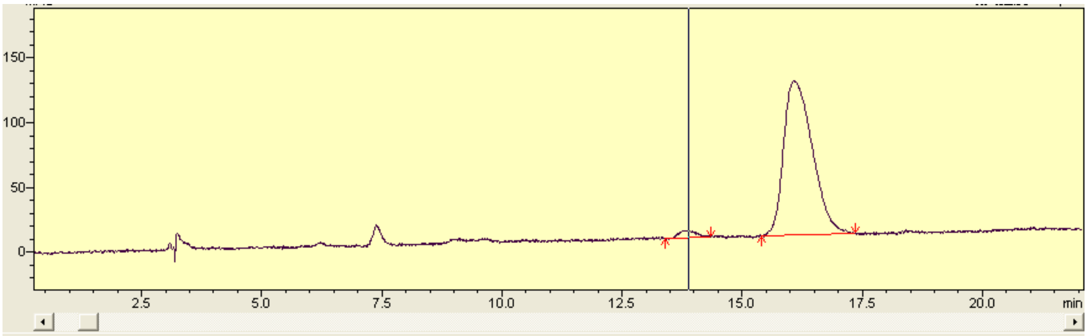

化合物表视图

ID#	名称	保留时间	峰#	面积	高度	面积%
1	RT13.890	13.841	2	143562	5805	2.8188
2	RT16.081	16.079	3	4946124	119356	97.1155

Run 5

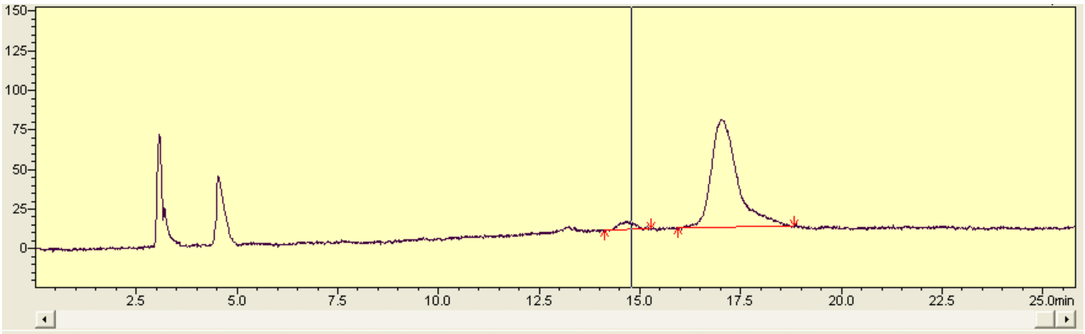

化合物表视图

ID#	名称	保留时间	峰#	面积	高度	面积%
1	RT14.777	14.681	1	133630	5152	4.1836
2	RT17.028	17.028	2	3060470	67960	95.8164

Run 1 with half catalyst mass

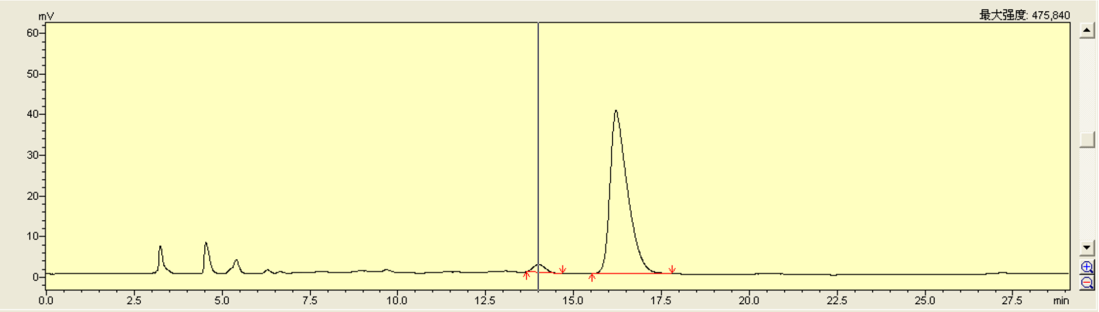

化合物表视图

ID#	名称	保留时间	峰#	面积	高度	面积%
1	RT14. 001	14. 001	1	46135	1885	3. 2230
2	RT16. 201	16. 201	2	1385280	40053	96. 7770

Run 2 with half catalyst mass

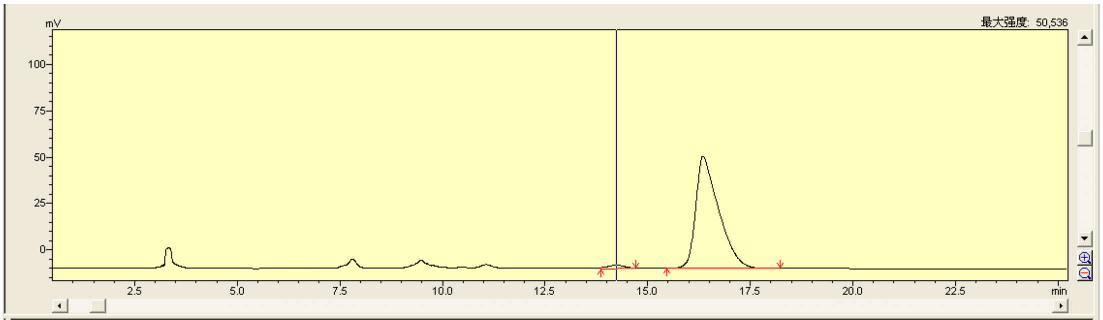

化合物表视图

ID#	名称	保留时间	峰#	面积	高度	面积%
1	RT14. 227	14. 227	1	61736	2000	2. 5573
2	RT16. 343	16. 343	2	2352396	60473	97. 4427

Run 3 with half catalyst mass

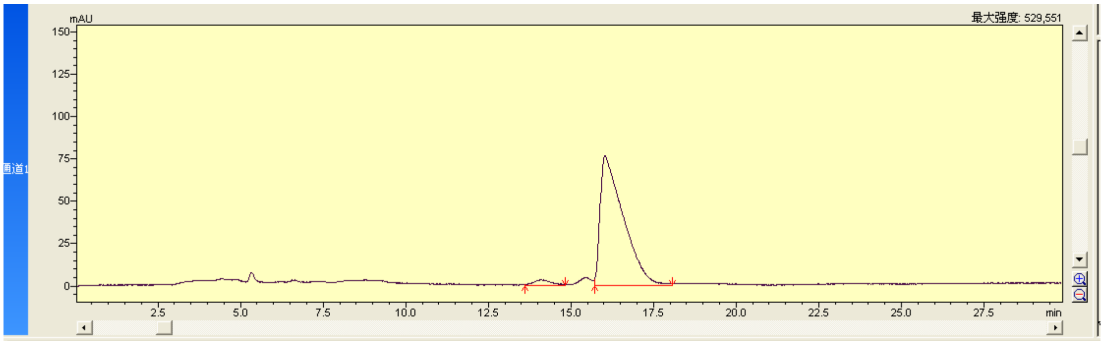

化合物表视图

ID#	名称	保留时间	峰#	面积	高度	面积%
1	RT14. 108	14. 108	1	109209	3135	2. 8671
2	RT16. 022	16. 022	2	3699827	76249	97. 1329

Table S4a. Reusability of catalyst **2** for the hydration/ATH one–pot Enantioselective cascade reaction of phenylacetylene.[a]

**Entry**	**1**	**2**	**3**	**4**	**5**
**Conv.**^{b} **[%]**	97	96	94	94	92
**ee**^{c} **[%]**	99	97	96	96	95

[a] Reaction conditions: 0.25 mmol of alkyne **1a**, cobaloxime (2 mol%) in MeOH (1 mL), heated at 65 ^{o}C under aerobic conditions, reaction time (5 h, quenched with 1 mL of H_2O; then adjust PH to 7 with 0.4 mol/L NaOH, **Catalyst 2** (20 mg, 1.04 μmol of Ru (0.4 mol%), based on ICP analysis), HCOONa (170 mg, 10 equiv), reaction temperature 35 ^{o}C, reaction time 12 h. ^{b} The Conv. were determined by ^{1}H NMR. ^{c} ee values were determined by chiral HPLC analysis.

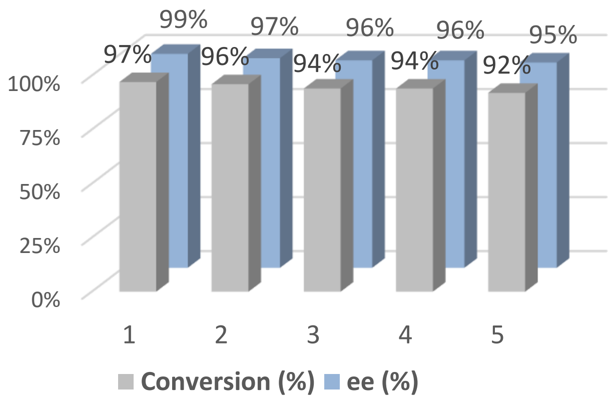

Table S4b. Reusability of catalyst **2** for the hydration/ATH one–pot Enantioselective cascade reaction of phenylacetylene.[a]

**Entry**	**1**	**2**	**3**
**Conv.**^{b} **[%]**	93	90	86
**ee**^{c} **[%]**	95	95	94

[a] Reaction conditions: 0.25 mmol of alkyne **1a**, cobaloxime (1 mol%) in MeOH (1 mL), heated at 65 ^{o}C under aerobic conditions, reaction time (5 h, quenched with 1 mL of H_2O; then adjust PH to 7 with 0.4 mol/L NaOH, **Catalyst 2** (10 mg, 0.52 μmol of Ru (0.2 mol%), based on ICP analysis), HCOONa (170 mg, 10 equiv), reaction temperature 35 ^{o}C, reaction time 12 h. ^{b} The Conv. were determined by ^{1}H NMR. ^{c} ee values were determined by chiral HPLC analysis.

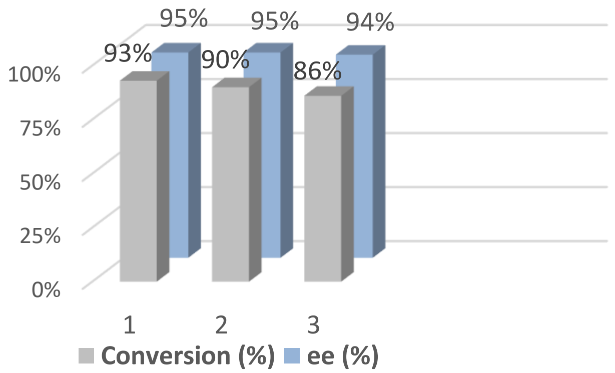

Figure S10. NMR spectra.

(*R*)-1- Phenethylalcohol

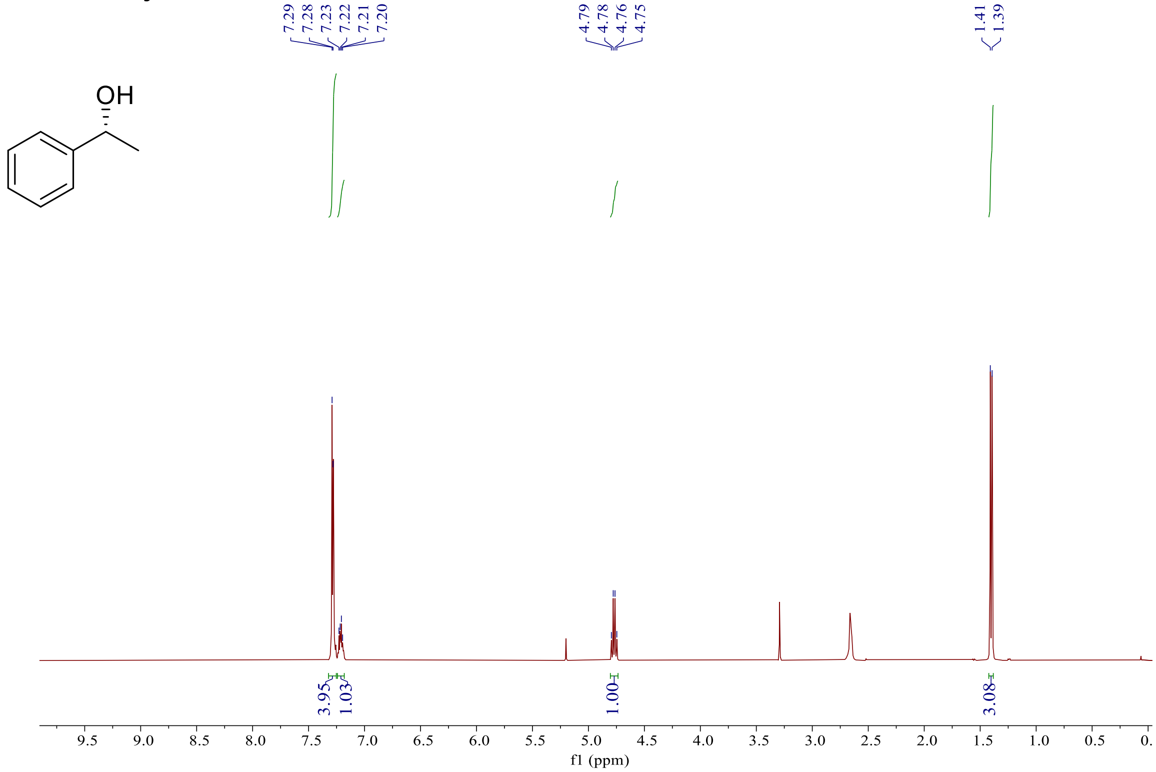

(*R*)-1-(p-tolyl)ethanol

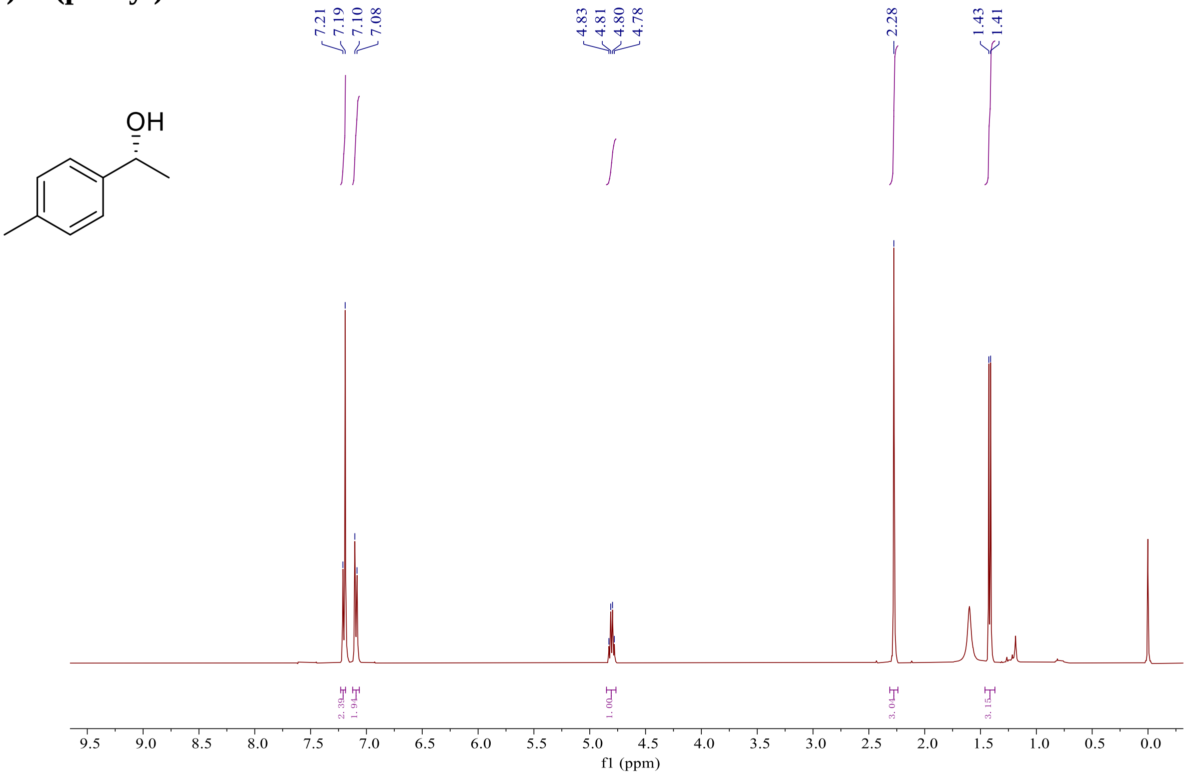

(*R*)-1-(4-ethylphenyl)ethano

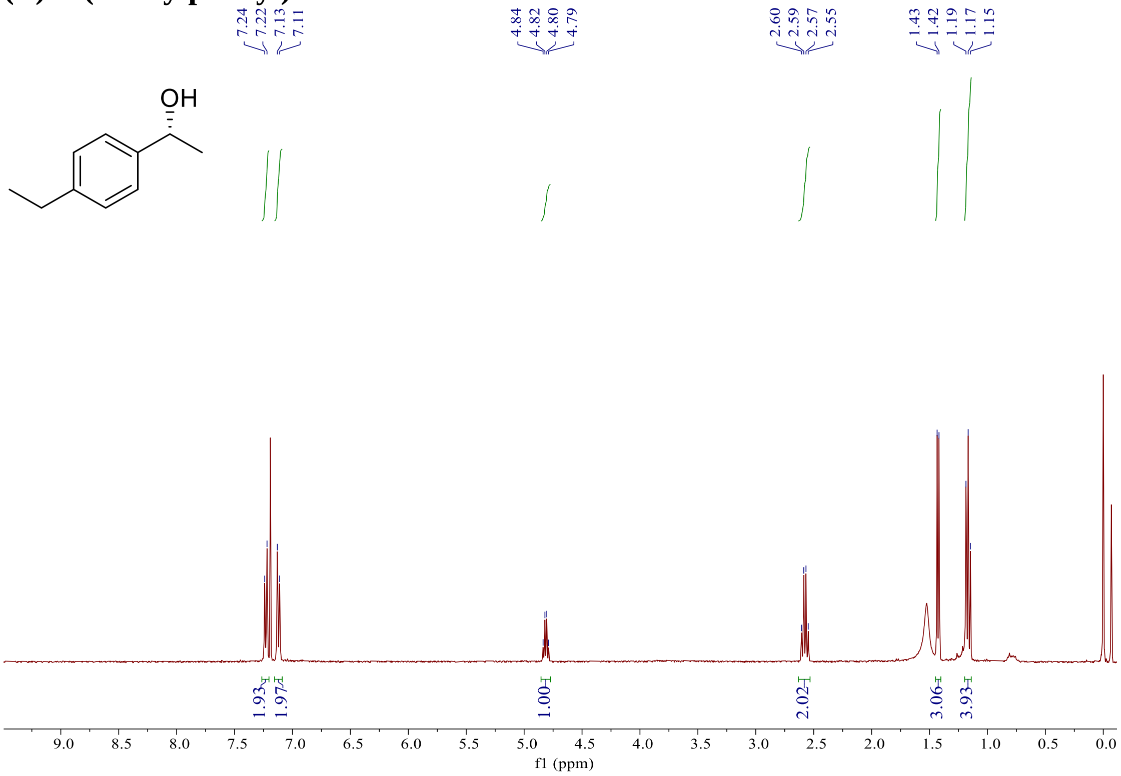

(*R*)-1-(4-propylphenyl)ethanol

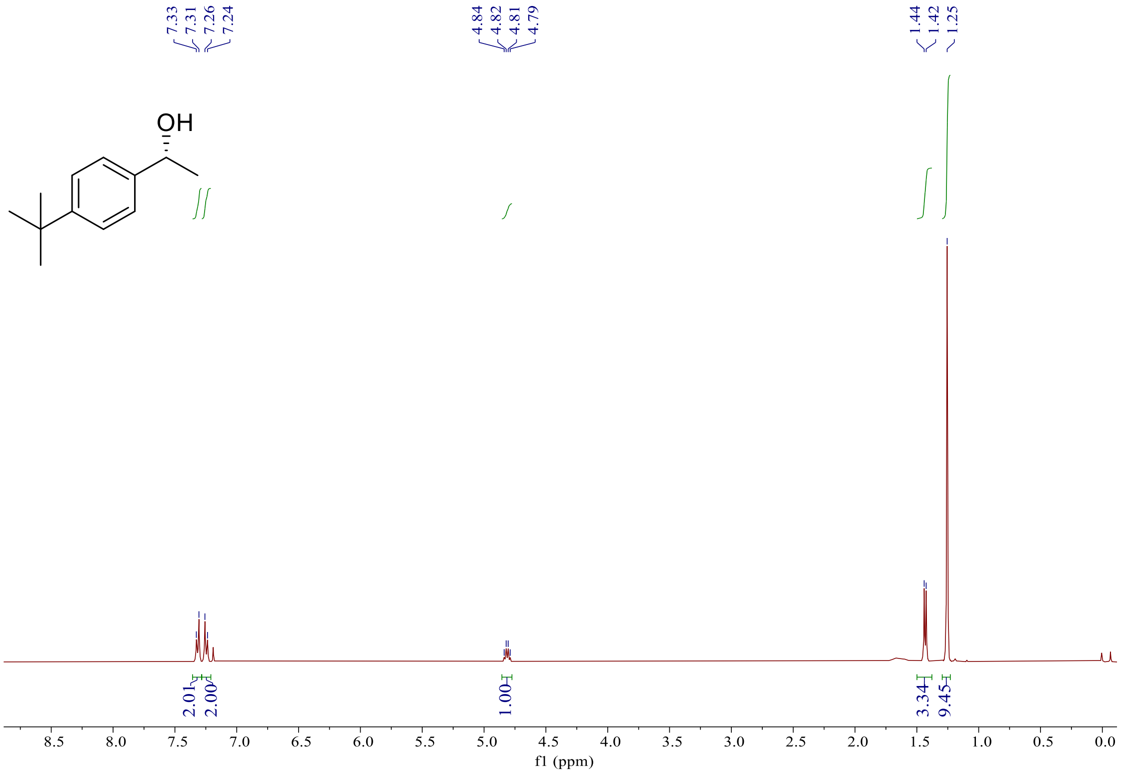

(*R*)-1-(4-methoxyphenyl)ethanol

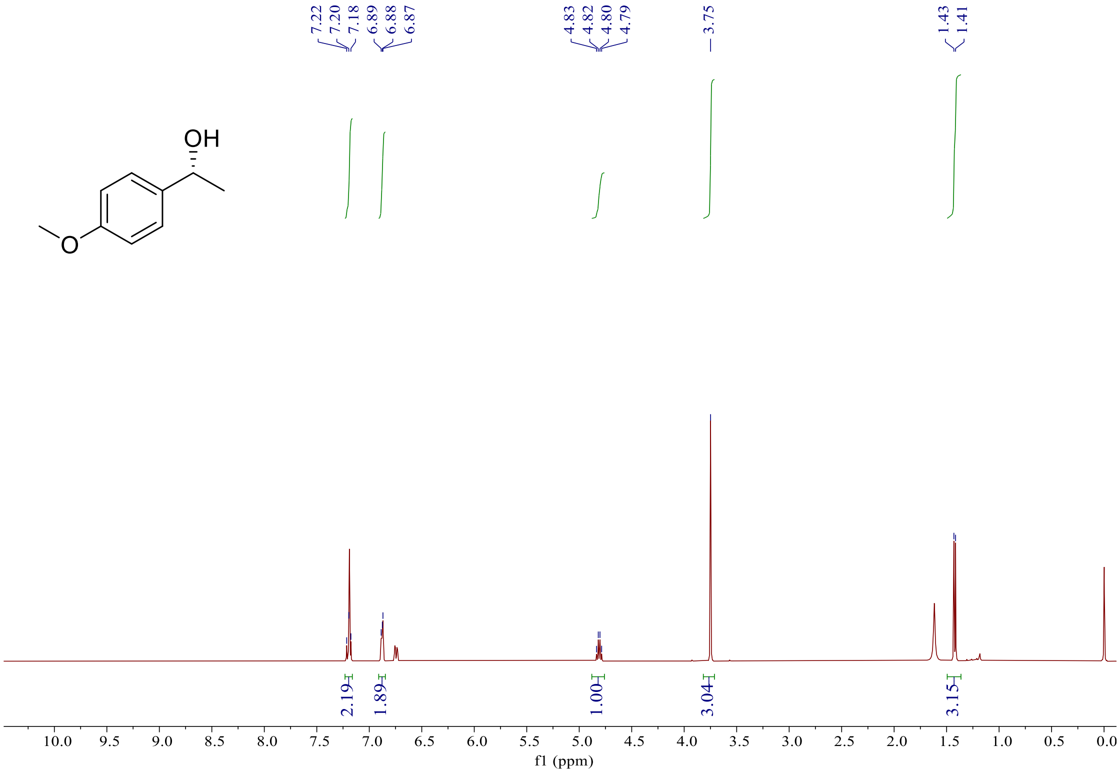

(*R*)-1-(2-methoxyphenyl)ethanol

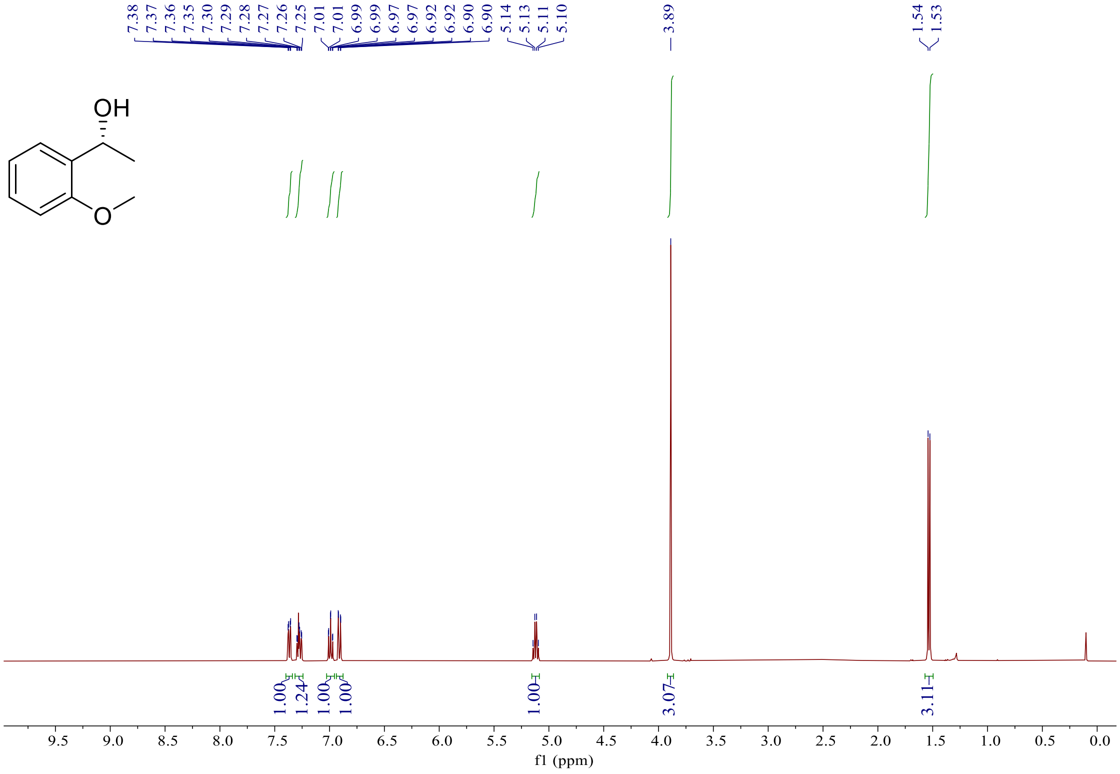

(*R*)-1-(3-methoxyphenyl)ethanol

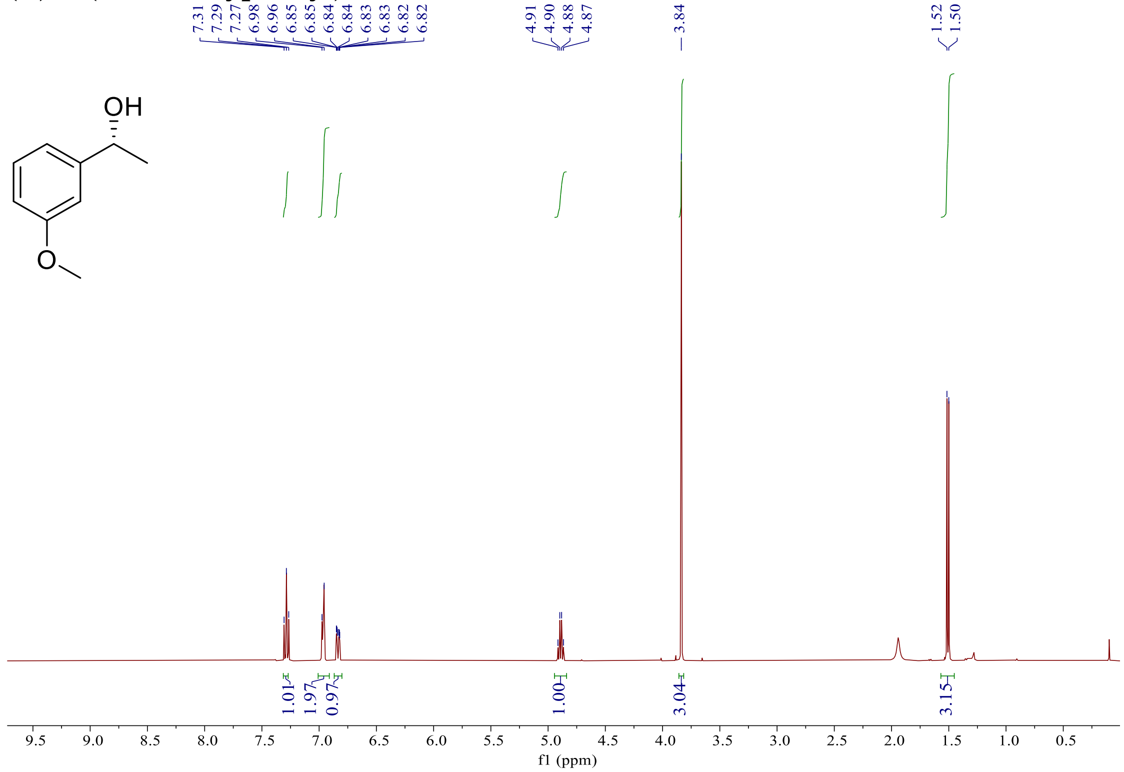

(*R*)-1-(4-fluorophenyl)ethanol

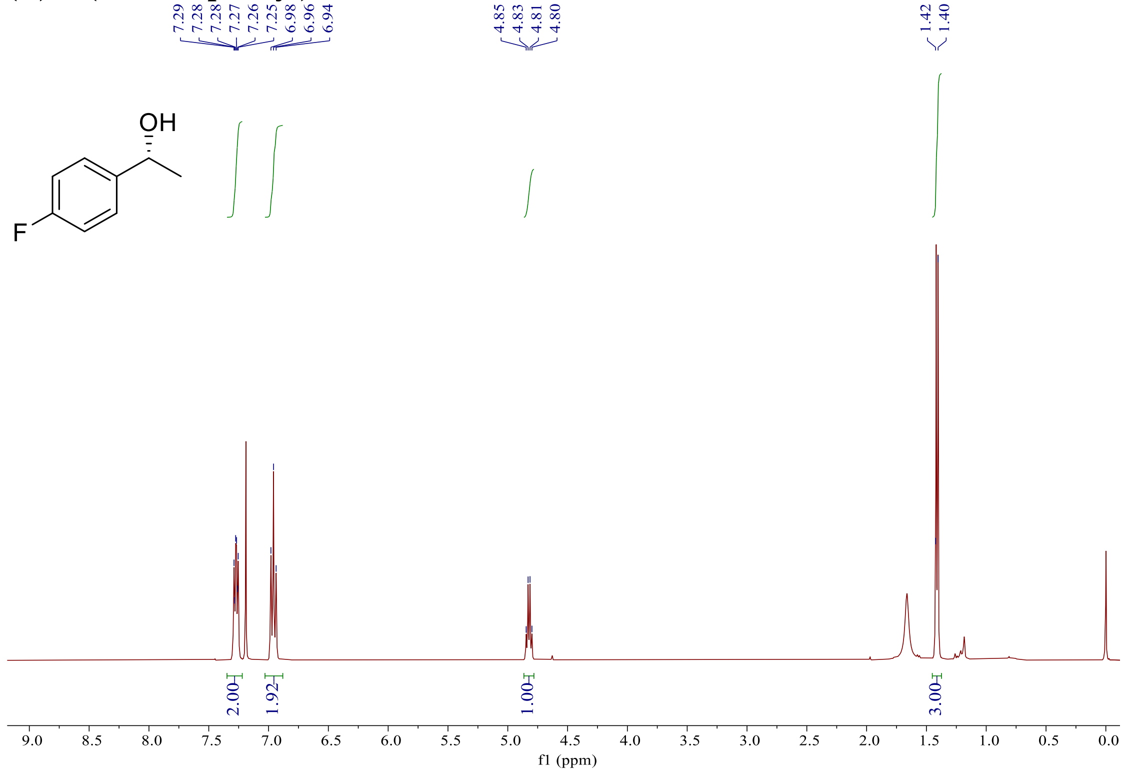

(*R*)-1-(4-bromophenyl)ethanol

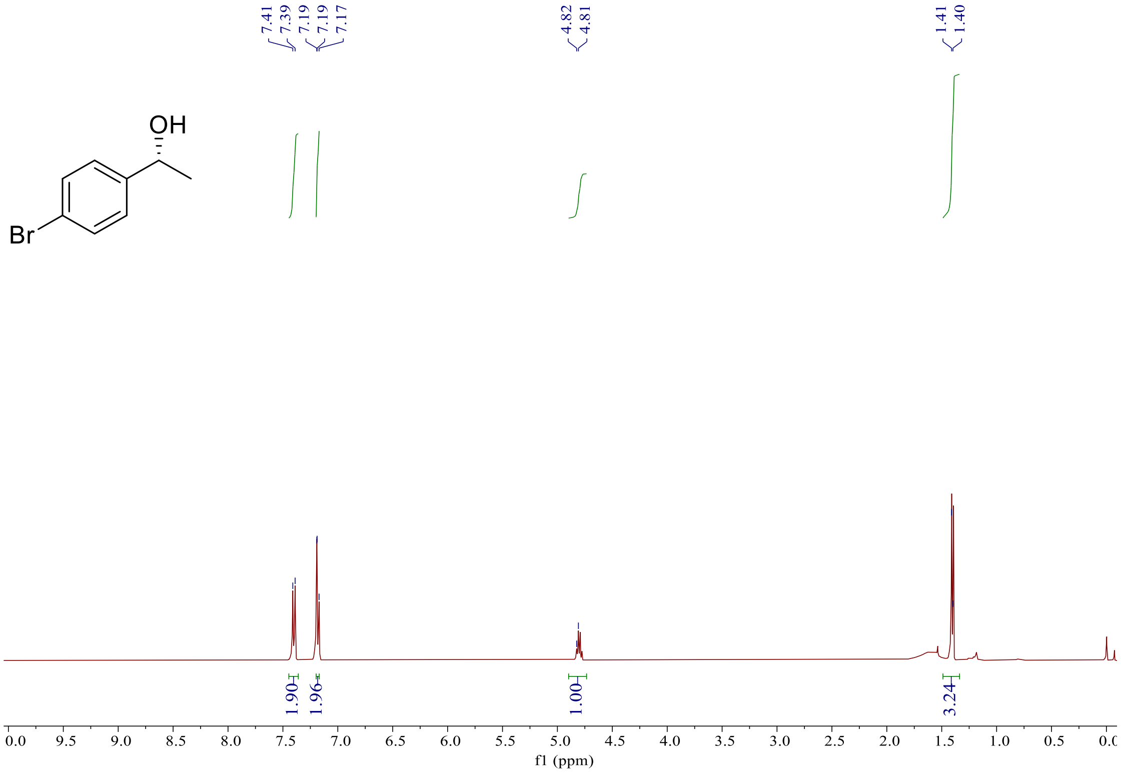

(*R*)-1-(4-chlorophenyl)ethanol

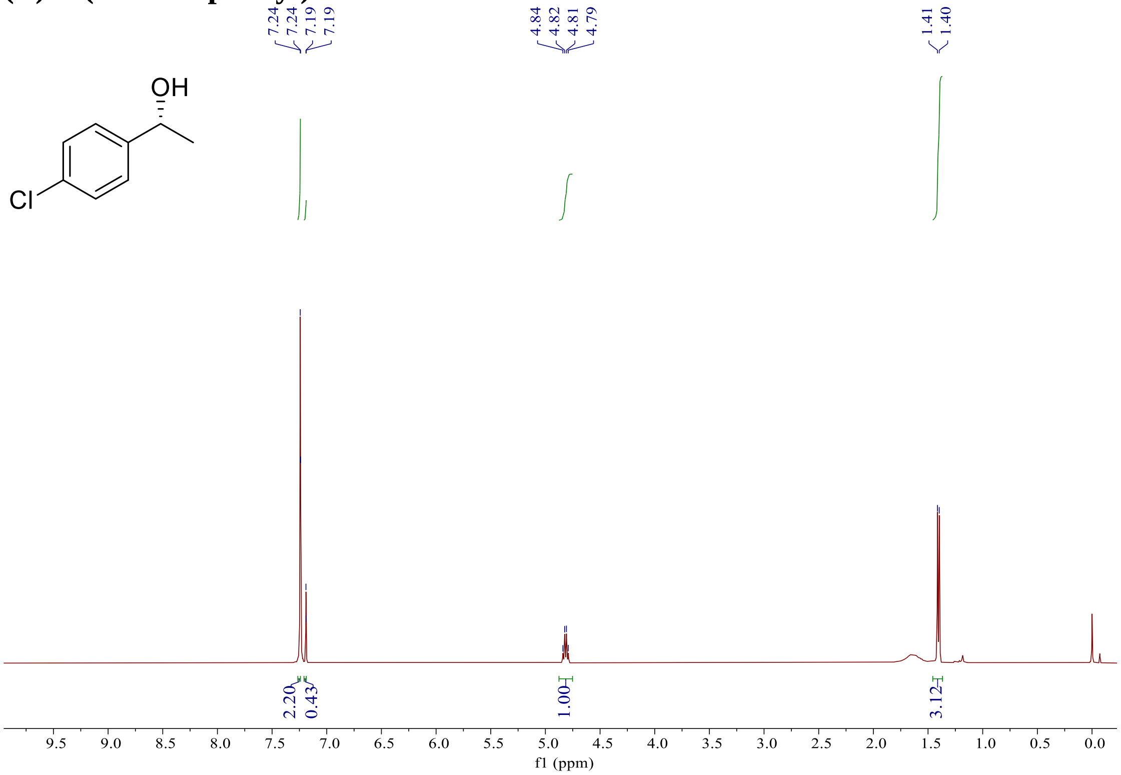

(*R*)-1-(4-nitrophenyl)ethanol

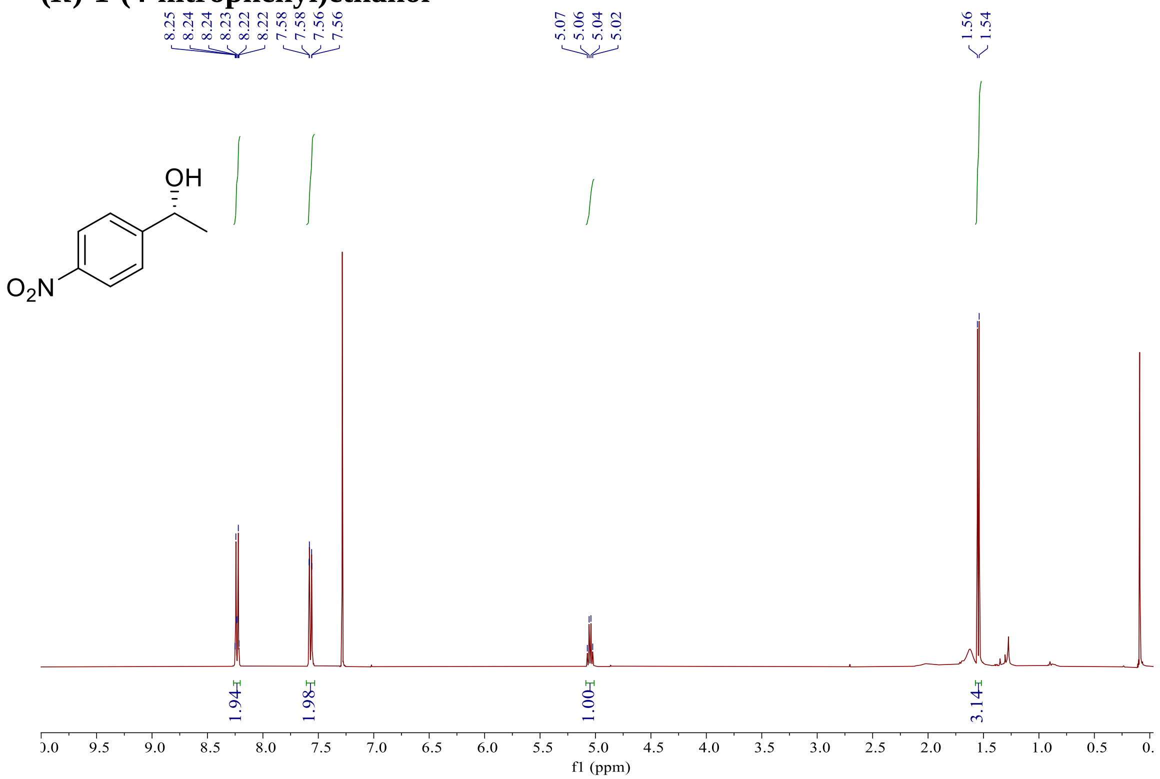

(*R*)-1-(3-bromophenyl)ethanol

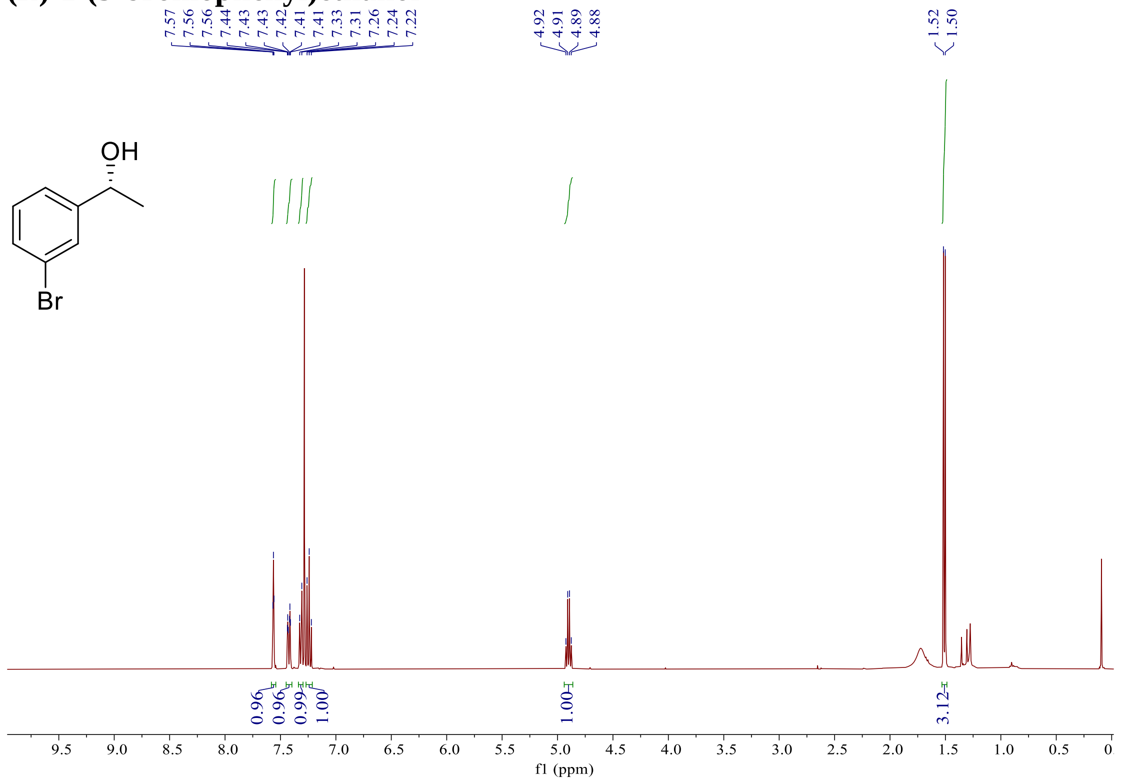

(*R*) -1-(2-Thiophen)ethanol

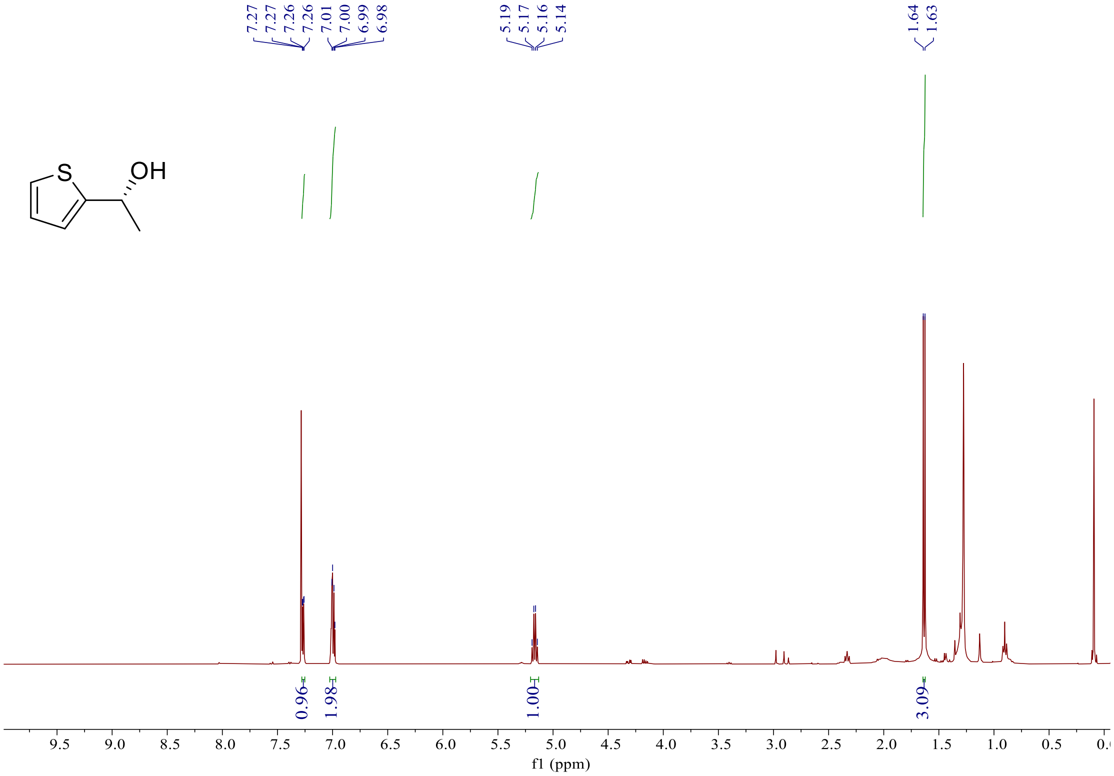

(1*R*,1'*R*)-1,1'-(1,4-phenylene)diethanol

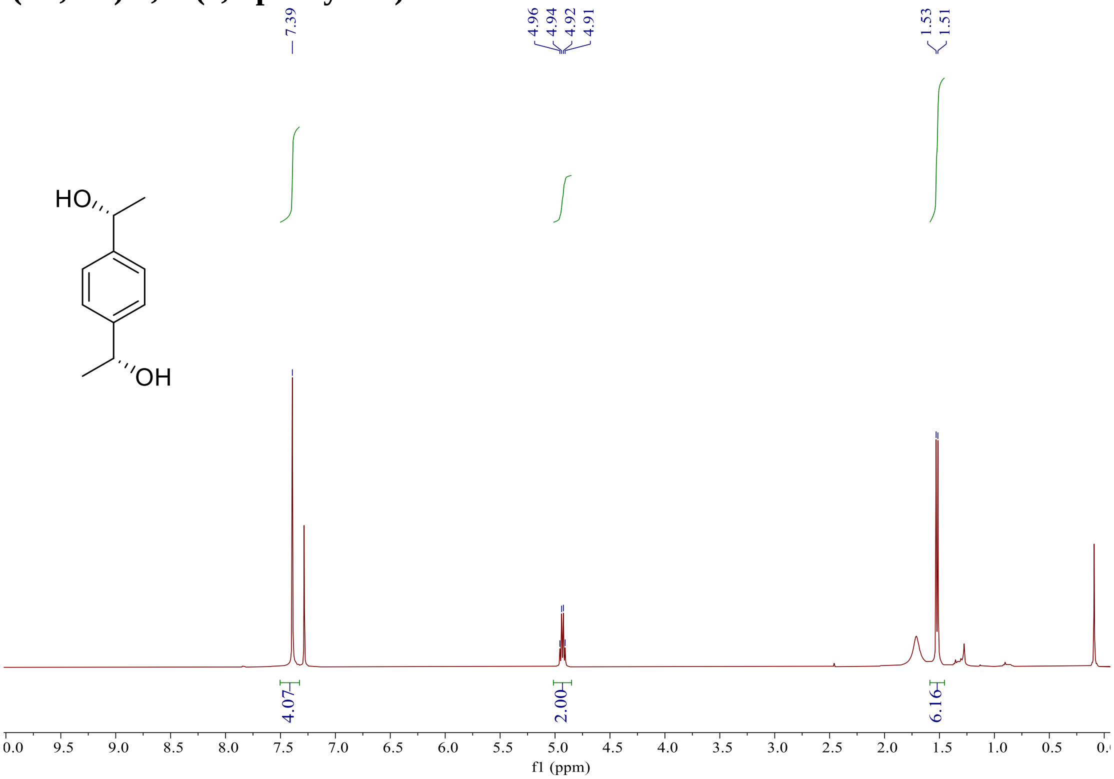

References

[1] Li, J., Wang, C., Xue, D., Wei, Y. and Xiao, J., (2013). One-pot transformation of alkynes into alcohols and amines with formic acid. *Green Chem.,* 15, 2685-2689. DOI: 10.1039/C3GC41133H.

[2] Li, F., Wang, N., Lu, L., Zhu, G., (2015). Regioselective hydration of terminal alkynes catalyzed by a neutral gold(I) complex [(IPr)AuCl] and one-pot synthesis of optically active secondary alcohols from terminal alkynes by the combination of [(IPr)AuCl] and Cp*RhCl[(*R*,*R*)-TsDPEN]. *J. Org. Chem.,* 80(7), 3538-3546. DOI: 10.1021/acs.joc.5b00164.

[3] Wang, S., Miao, C., Wang, W., Lei, Z., Sun, W., (2014). A salen–Co^{3+} catalyst for the hydration of terminal alkynes and in tandem catalysis with Ru–TsDPEN for the one-pot transformation of alkynes into chiral alcohols. *ChemCatChem* 6, 1612-1616. DOI: 10.1002/cctc.201400071.

[4] Lu, J., Dimroth, J., Weck, M., (2015). Compartmentalization of incompatible catalytic transformations for tandem catalysis. *J. Am. Chem. Soc.,* 137(40), 12984-12989. doi: 10.1021/jacs.5b07257.

[5] Liu, S., Liu, H., Zhou, H., Liu, Q. and Lv, J., (2018). Transformation of alkynes into chiral alcohols via TfOH-catalyzed hydration and Ru-catalyzed tandem asymmetric hydrogenation. *Org. Lett.,* 20(4), 1110-1113. DOI: 10.1021/acs.orglett.8b00034.

[6] Liu, H., Liu, S., Zhou, H., Liu, Q. and Wang, C., (2018). One-pot synthesis of chiral alcohols from alkynes by CF_3SO_3H/ruthenium tandem catalysis. *RSC Adv.*, 8, 14829-14832. DOI: 10.1039/C8RA02224K.
